# Hybrid MXene coatings: unlocking synergistic lubrication properties of Ti_3_C_2_T_x_ and Nb₂CT_x_ MXenes for improved tribological performance

**DOI:** 10.1038/s41598-025-32533-6

**Published:** 2025-12-22

**Authors:** Christina Danecker, Sabine Schwarz, Marko Piljevic, Jakob Rath, Martin Nastran, Bernhard C. Bayer, Michael Naguib, Ahmad Majed, Karamullah Eisawi, Pierluigi Bilotto, Carsten Gachot

**Affiliations:** 1 Institute of Engineering Design and Product Development, Research Unit Tribology, TU Wien, Lehargasse 6, Building BA, 9th floor, Vienna, 1060 Austria; 2 University Service Center for Transmission Electron Microscopy (USTEM), TU Wien, Stadionallee 2, Vienna, 1020 Austria; 3 AC2T research GmbH, Viktor-Kaplan-Straße 2/C, Wiener Neustadt, 2700 Austria; 4 Centre for Electrochemical Surface Technology, CEST GmbH, Wiener Neustadt, A-2700 Austria; 5 Analytical Instrumentation Center, TU Wien, Lehargasse 6, Vienna, 1060 Austria; 6 Institute of Materials Chemistry, TU Wien, Getreidemarkt 9, Vienna, 1060 Austria; 7 School of Science and Engineering, Tulane University, 208 Stanley Thomas Hall, New Orleans, US

**Keywords:** Engineering, Materials science

## Abstract

The development of advanced solid lubricants is critical for enhancing energy efficiency and durability in mechanical systems. In this study, we investigate the tribological performance of hybrid solid lubricant coatings composed of two-dimensional titanium carbide (Ti₃C_2_T_x_)- and niobium carbide (Nb₂CT_x_)-based MXenes. Coatings were applied via spray deposition onto AISI 304 stainless steel substrates and tested under dry sliding conditions against Al₂O₃ counterbodies. While individual MXene coatings exhibited limited friction stability, the hybrid Ti₃C_2_T_x_/Nb₂CT_x_coating demonstrated a significantly reduced and stable coefficient of friction (COF < 0.2) throughout the test duration. Comprehensive surface and structural analyses of the wear tracks including SEM-EDS, Raman spectroscopy, and TEM revealed the formation of a compact, stratified tribofilm. We propose as a phenomenological model that under tribological stress, the hybrid system undergoes adaptive reconfiguration: Ti₃C_2_T_x_ anchors to the substrate, enhancing adhesion and mechanical integrity, while Nb₂CT_x_ migrates to the sliding interface, acting as a sacrificial layer. This dynamic redistribution results in a synergistic interaction that enhances tribochemical resilience and wear resistance. These findings establish hybrid MXene coatings as a promising strategy for engineering next-generation solid lubricants, offering new pathways for the design of high-performance, energy-efficient coatings in demanding industrial applications.

**Supplementary Information:**

The online version contains supplementary material available at 10.1038/s41598-025-32533-6.

## Introduction

The transition to sustainable energy production and transportation is essential for reducing emissions and addressing climate change. Securing energy efficiency is equally important in the endeavour of achieving sustainability goals. The International Energy Agency (IEA) highlights that about 30% of cumulative CO_2_emission reductions can be achieved by promoting energy efficiency in technologies and the adoption of less energy-intensive materials and processes^1^.

Tribology, the science of friction, wear and lubrication, plays a crucial role in enhancing the efficiency, reliability, and lifespan of mechanical systems. Indeed, tribological losses are responsible of approximately 23% of global energy consumption. Therefore, with almost all industry sectors depending on machines with moving components, reducing friction-related losses is fundamental to improving overall performances^2^.

As friction and wear lead to considerable material and energy losses in mechanical systems^3^, the simplest and most efficient solution is the application of a lubricant to separate the interacting surfaces^4^. Depending on the application and environmental conditions, various lubricants are used to reduce friction and wear, such as mineral- or synthetic oils^5^, greases^6^, ionic liquids^7^, organic lubricant (e.g., Polytetrafluoroethylene - PTFE), and solid lubricants^8^. PTFE is able to reduce significantly the coefficient of friction of sliding surfaces and can be easily applied as coating, however, PTFE has poor thermal management characteristics, making it not ideal for applications where large amount of heat is transferred^9^. Moreover, the recent EU regulations place a lot of attention on PTFE-based products, motivating the research for valid alternatives for tribological applications. Thus, as technological and industrial needs become more demanding for high performances in complex scenarios (e.g., aerospace), the scientific research stirred toward evaluating the tribological performances of novel advanced materials.

Among these, two-dimensional (2D) materials with layered structures have emerged as a highly researched class of solid lubricants, due to their exceptional mechanical, physical, chemical, and tribological properties^10–12^. 2D materials are thin, sheet-like structures that consist of one or a few atomic layers in thickness^12^. These layered materials can form easy-to-shear tribolayers, which separate sliding surfaces and help reduce friction and wear^13^. The functionality of 2D materials is based on weak van der Waals interactions between the layers, enabling them to slide under low shear forces. At the same time, strong in-plane covalent bonds hold the atoms together, allowing the layers to move easily without separating completely^14^. Molybdenum dioxide (MoS_2_) is the benchmark for solid lubrication, especially when referring to challenging ambient such as space applications. However, it is now well known that any environmental changes deteriorate significantly the tribological performance of MoS_2_, which cannot rely on facile functionalization as they do not present surface termination groups^15^. In contrast, MXenes are a family of 2D materials presenting important surface termination, making them possible candidates to resolve the open challenges in solid lubrication such as environmental stability and durability (wear life)^16–18^.

MXenes, 2D transition metal carbides, nitrides, and carbonitrides, have recently received considerable attention^19^. They are produced by removing the A-group layer atoms (e.g. Al) from M_*n*+1_AX_n_ or MAX phases^20^. After removing the A group elements, multilayered MXenes remain, described by the chemical formula M_*n*+1_X_n_T_x_ (*n* = 1 to 4). The surface is terminated with functional groups such as -O, -OH, -F and/or -Cl, collectively represented as ‘T_x_’, depending on the specific etching route used (HF, MILD method, Electrochemistry or Molten Salt)^4,19,21,22^. MXenes demonstrate application potential in several fields, such as energy storage^23,24^, catalysis^24^ biomedical applications for biosensors^25^ or drug delivery^26,27^. One of the most well-known and extensively studied MXene is titanium carbide (Ti₃C_2_T_x_), produced by selective etching of Ti₃AlC₂. Its properties include high electrical conductivity^28^, biocompatibility^29^, antibacterial activity^30^ and good mechanical characteristics such as tensile strength, elastic modulus, and fracture strain^31^. Due to their chemical and structural versatility combined with their easy-to-shear ability and strong interfacial bonding, Ti₃C_2_T_x_ have emerged as a promising material for tribological applications^16,17^, including their use as lubricant additives^32,33^ and solid lubricant coatings^18,34^.

Another notable member of the MXene family is niobium carbide (Nb₂CT_X_)^35^. Nb₂CT_X_ has been used in various energy applications due to its exceptional electrical conductivity, outstanding chemical stability and high electronic conductivity^36^. These applications include supercapacitors^37^, rechargeable batteries^35^, sensors^38^, and even biomedical uses^39^.

Beyond energy-related applications, recent studies have also explored the tribological potential of Nb based MXenes. By using Atomic Force Microscopy (AFM), it was observed that Nb₂CT_X_ MXenes exhibited lower friction and adhesion forces compared to Ti₃C_2_T_x_, with both properties decreasing as the temperature increased^40^. The different behaviours in friction and adhesion were attributed to differences in surface dipole moment density, with Nb₂CT_X_ having a denser surface compared to Ti₃C_2_T_x_^40^.

Nb₂C nanosheets with varying degrees of oxidation were applied as solid lubricant additives in water-based lubrication systems^41^. The results showed that a medium oxidation degree significantly reduced the coefficient of friction by 90.3% and the wear rate by 73.1% compared to pure water, demonstrating the added value of Nb₂C^41^.

These exceptional tribological characteristics of MXenes (Ti₃C_2_T_x_ and Nb₂CT_X_ alike) are strongly influenced by factors such as the number of nanosheets, the orientation of individual monolayers, the lateral flake size, the film thickness, the interlayer interactions, the substrate interaction, and surface terminations^8,31,42^. The latter, directly affect the adhesion of both the individual layer and at the interface with the substrate, as presented in a recent density functional theory (DFT) study taking iron and iron oxide as substrates^43^. DFT suggests that tuning the density of -OH and -F terminations may lead to a significant improvement in the friction and wear behaviour of MXenes^43^, as proven in our recent work on the tribological performances of electrochemically synthesized MXenes^44^.

The oxidation properties of MXenes in air, liquid, and solid environments reveals their strong susceptibility to degradation as a key challenge. For instance, Ti₃C_2_T_x_ flakes in liquids is prone to form TiO_2_, which reduces conductivity and limits long-term stability^45^. Similarly, at 20% relative humidity, the coefficient of friction of Ti₃C_2_T_x_ remains stable for about 4000 sliding cycles, whereas at higher humidity levels (approximately 80%), a significant rise in the coefficient of friction is observed^46^.

Novel strategies are required to overcome specific challenges in MXene tribology such as oxidation stability and tribochemical resilience. The exploration of MXene hybrids is gathering interest owing to the possibility of igniting synergistic effects that may overcome limits of the singular species. For instance, a recent study has shown the ability to tailor the optical properties of Ti₃C_2_T_x_ and Nb₂CT_X_hybrids^47^. Similarly, Ti₃C_2_T_x_ combined to MoS_2_ has demonstrated great tribological performances, with a coefficient of friction as low as 0.14 and a wear rate of 0.49 × 10–5 mm³/Nm^48^.

Inspired by these works, hereby we investigate the synergistic effect of combining Ti₃C_2_T_x_ and Nb₂CT_X_ to form hybrids with advanced tribological performances.

Specifically, we evaluate the tribological performance of a spray coated Nb₂CT_X_/Ti₃C_2_T_x_ hybrid deposited onto AISI 304 steel substrates, which are then tested under linear sliding conditions against a Al_2_O_3_ counterbody. Uncoated AISI 304 steel substrates, Ti₃C_2_T_x_, and Nb₂CT_X_ coatings were used as reference materials. Detailed post-experimental analysis of the coatings and the corresponding wear tracks provides insight into the underlying mechanisms of the observed tribological behaviour. The hybrid coating exhibited a stable coefficient of friction (COF) below 0.2, demonstrating excellent lubricity. This is attributed to the tribo-induced formation of a compact, patchy tribofilm, which effectively reduces the COF and protects the substrate from wear. Surface analysis of the wear tracks was conducted to investigate the underlying tribochemical processes, using techniques such as Raman spectroscopy, X-ray photoelectron spectroscopy (XPS), and transmission electron microscopy (TEM).

In conclusion, we demonstrate that the Nb₂CTX/Ti₃C_2_T_x_ hybrid coating provides superior tribological performance due to the synergistic interaction between the two MXene species. Under sliding conditions, Nb₂CT_X_ and Ti₃C_2_T_x_ undergo adaptive reconfiguration aimed at minimizing the COF. Nb₂CT_X_ becomes more exposed to the sliding interface, acting as a sacrificial layer that undergoes oxidation, while Ti₃C_2_T_x_ preferentially positions itself at the interface with the substrate, enhancing adhesion and contributing to the mechanical robustness of the tribofilm. This dynamic redistribution underscores the complementary roles of the two MXenes in forming a stable, low friction tribolayer.

Our findings present a novel strategy for engineering MXene-based solid lubricant hybrid composites; wherein two-dimensional materials are designed to exhibit enhanced functional behaviour. This methodology leverages the vast compositional diversity of the MXene family and aligns with the growing interest in high-entropy systems. As such, it opens new avenues for both fundamental research in advanced materials and the development of innovative, application-driven solutions for industry.

## Materials and methods

### Synthesis of Ti_3_AlC_2_ and Nb_2_AlC MAX phases

Elemental powders of titanium (Ti, −325 mesh, 99%, Thermo Scientific), aluminum (Al, −325 mesh, 99.5%, Thermo Scientific), and graphite (C, 7–11 μm, 99%, Alfa Aesar) were combined in a Ti: Al: C molar ratio of 3.00:1.20:1.88. The mixture was placed in a high-density polyethylene (HDPE) container along with ten 10 mm yttria-stabilized zirconia (YSZ) balls and mixed using a Turbula T2F mixer at 56 rpm for 3 h. It was then transferred to an alumina crucible and heated in a tube furnace under a continuous argon flow (0.2 L/min). The temperature was increased to 1600 °C at a rate of 5 °C/min and held for 2 h. After the reaction, the system was allowed to cool naturally to room temperature under inert atmosphere. The resulting lightly sintered Ti_3_AlC_2_ brick was ground and sieved to obtain powders smaller than 45 μm. Synthesis of Nb_2_AlC MAX phase followed the same procedure however using elemental powders of niobium, aluminium, and graphite with molar ratios Nb: Al: C of 2.00:1.30:0.95 C. After mixing, the mixture was pressed into green pellets 12–13 g/pellet. The pellets were sintered at 1600 °C and held for 4 h.

### Synthesis of Ti_3_C_2_T_x_ and Nb_2_C_x_ MXene phases

Ti₃C_2_T_x_ was obtained by selectively etching the aluminum layers from Ti_3_AlC_2_ using an in-situ HF-generating etchant composed of potassium fluoride (KF, 99%, Thermo Scientific) and hydrochloric acid (HCl, 9 M, Fisher Chemicals). The etching solution was prepared by mixing 20 mL of 9 M HCl per gram of Ti₃AlC₂ with KF at a molar ratio of 9:1 (KF: Ti₃AlC₂). Ti₃AlC₂ powder was gradually added to the etchant under magnetic stirring at 500 rpm and maintained at 45 °C for 48 h. Following etching, the product was soaked in 9 M HCl for 18 h to remove excess salts. The resulting powder was then washed by distributing it into 50 mL centrifuge tubes, using one tube per 0.5 g of precursor. Deionized (DI) water was added, followed by centrifugation at 3500 rpm for 2 min. The supernatant was discarded, fresh DI water was added, and the sediment was redispersed using a vortex mixer. This process was repeated until the pH of the supernatant exceeded 6. Nb_2_C_x_ was produced by inserting Nb_2_AlC powders into hydroflouric acid (HF, 48–51%, Alfa Aesar) with a ratio of 1 g: 20 ml the sample was stirred at 40 °C for 5 days. The washing and filtering steps were like that of Ti₃C_2_T_x_.

The morphology of the as-synthesised Ti₃C_2_T_x_ and Nb₂CT_X_ MXenes was characterised by scanning electron microscopy (SEM, FEI Quanta 250 FEG) operated at an acceleration voltage of 5 kV. Secondary electron imaging was performed using an Everhart-Thornley detector (ETD). In addition, TEM (Tecnai F20) was performed at 200 kV acceleration voltage for higher-resolution imaging.

In addition, X-ray diffraction (XRD) and Raman spectroscopy were performed on both MXene powders to gain further information about their surface terminations. XRD were carried out using a Malvern Panalytical X’Pert MPDII equipped with a X’Celerator semiconductor detector. Powder X-ray diffraction (PXRD) measurements were performed on a Θ/Θ Bragg–Brentano diffractometer equipped with a copper anode (Cu Kα radiation, λ = 1.5406 Å). Diffraction patterns were recorded over a 2θ range of 5° to 70°, with a total scan duration of 20 min.

A LabRam Aramis Raman microscope (Horiba Jovin Yvon, Germany) is used to record Raman spectra of Ti₃C_2_T_x_ and Nb₂CT_X_ MXene powder. Red raman laser with wavelength of 632 nm and laser power of 2 mW is used to excite samples. Spectra were recorded from 100 to 2000 cm^− 1^ with a 1200 mm^− 1^ grid. Both powders are tested separately before applying on substrate. Data are evaluated in OriginPro 2023b software, where background is removed and peaks are normalized.

### Coating deposition

Overall, four different samples were tested: one uncoated reference and three distinct coating systems — a pure Ti₃C_2_T_x_ coating, a Nb₂CT_X_ coating, and a hybrid coating composed of a combination of both MXenes. For the hybrid coating Nb₂CT_X_ were first applied, followed by the same amount of Ti_3_C_2_T_x_, ensuring a uniform mixture of both materials. These coatings were applied to mirror-polished stainless-steel platelets (AISI 304) with dimensions of 15 × 15 × 2 mm³, a Young’s modulus of 200 GPa, a Poisson´s ratio of 0.29, and an initial roughness S_q_ of about 0.18 μm. The solid lubricant coatings were deposited onto these substrates by using the airbrush spray-coating process. Uncoated plates and balls were also tested as reference materials. All MXenes were dispersed separately in ethanol at a concentration of 2 mg/mL, regardless of the composition (Table 1).

**Table 1:** Overview of the deposited coatings, including their design, thickness and applied volume.

Name	Material/Coating	Spray volume (mL)	Thickness tribofilm (nm)
reference	AISI 304	-	-
Ti_3_C_2_T_x_	MXene Ti_3_C_2_T_x_	4	1383.2 ± 142
Nb_2_CT_x_	MXene Nb_2_CT_x_	4	202.1 ± 56
Hybrid	Combination of Nb_2_CT_x_ + Ti_3_C_2_T_x_	4 (2 + 2)	230.5 ± 82

The following steps were taken to prepare the dispersion. The dispersions were first homogenised using a shear mixer (T25 basic IKA^®^ ULTRA-TURRAX^®^) for 5 min at 11,000 rpm. To improve the dispersion and distribution of the nanosheets, tip sonication (QSONICA CL-18 Sonicators) was applied for 1 h (5 s on, 5 s off, 50 W power), followed by 2 h of bath sonication. Directly afterwards, 4 mL of the dispersion was transferred via a syringe into a self-build spray coating set-up. The distance between nozzle and steel substrate was maintained at 10 cm, and the air pressure during spray-coating at 1,5 bar. To ensure rapid solvent evaporation, prevent droplet formation, and achieve uniform coatings the substrates were pre-heated and placed on a heating plate set to 80 °C during deposition.

To fabricate the hybrid coating, 2 mL of each MXene dispersion were sprayed onto the substrate, starting with Nb₂CT_x_ followed by Ti₃C_2_T_x_, resulting in a total spray volume of 4 mL. To prevent cross-contamination and ensure material purity, two identical airbrushes of the same model were used - one for each dispersion.

### Tribological experiments

The tribological performance of the different solid lubricant coatings was evaluated using a ball-on-disc tribometer (Rtec Instruments, MFT-2000 A) in linear sliding mode. The counterbody material was Al_2_O_3_ with a diameter of 6 mm. The linear sliding velocity was set to 1 mm/s, stroke length to 1 mm, the normal force to 0.25 N with a calculated hertzian contact pressure of 0.48 GPa, which is in the operating range of many machine elements such as roller bearings, and the test duration to 30 min. A case of moderate contact stresses is reflected in the initial contact pressure at the contact centre. The tests were carried out at room temperature between 20.2 °C and 23.5 °C and the relative humidity ranged between 20.5% and 25%. All experiments were performed three times to ensure reproducibility and statistical significance, as well as to calculate the mean values and error bars.

### Surface characterization of the wear track

Before and after tribological experiments, all samples were analysed by confocal laser scanning microscopy (CLSM, Keyence VK-X1100).

Surface morphology and wear tracks were analysed using a SEM equipped with an ETD detector. Imaging was carried out in conjunction with the preparation of cross sections and TEM lamellae prepared using a ThermoFisher Scios II dual beam system.

Surface sensitive chemical characterisation was carried out using X-ray photoelectron spectroscopy (XPS, PHI Versa Probe III-spectrometer) equipped with a monochromatic Al-Kα X-ray source and a hemispherical analyser (acceptance angle: ±20°). Pass energies of 140 eV and 27 eV and step widths of 0.5 eV and 0.05 eV were used for survey and detail spectra, respectively. (Excitation energy: 1486.6 eV Beam energy and spot size: 2 W onto 100 μm; Mean electron takeoff angle: 45° to sample surface normal; Base pressure: 5 × 10^− 10^ mbar, Pressure during measurements: <1 × 10^− 8^ mbar). Samples were mounted on non-conductive tape. A combination of electronic and ionic charge compensation was used for all measurements (automatized as provided by PHI). Surface cleaning was performed by using a PHI Gas Cluster Ion Gun (GCIB) (5 kV for 300 s). A spot size of 20 μm is used to perform XPS analysis of the wear track. The smallest accessible spot size is 9 μm diameter. Spectral analysis was carried out using CasaXPS, with corrections for instrument transmission and background removal via the Shirley background^49^ and sensitivity factors provided by PHI^50^. The calibration of the XPS spectra was done by selecting the C1s peak and shifting the C-C and C-H- component to a value of 248.8 eV. The binding energy (BE) scale and intensity were calibrated by using methods described in ISO15472, ISO21270, and ISO2437. Deconvolution of spectra was carried out by using a Voigtian lineshape if not stated otherwise. All content values shown are in units of relative atomic percent (at.-%), where the detection limit in survey measurements usually lies around 0.1–1 at.- %, depending on the element. Assignment of different components was primarily done using Refs^51,52^.

Besides the powder characterization, Raman spectroscopy was employed to characterize the coatings and wear tracks. Hybrid MXenes wear scars were recorded in three different zones: reference coating, tribolayer and pile-up zone. Both wear scars of MXenes tested separately were recorded in two zones: tribolayer and pile-up zone. Settings used during measurement were: RTD exposure time of 1 s, exposure time of 100 s and Accumulation number of 2. All examined zones were measured without filter using a 50x Fluotar objective.

The wear tracks were further analysed by scanning electron microscopy (SEM, Zeiss Eco 10, equipped with a tungsten cathode as an electron source) in combination with EDS. A Zeiss SmartEDS detector (silicon drift with a silicon nitride window) was employed for EDS measurements. The excitation energy was 20 kV, and the base system pressure was 1,3 × 10^− 4^ mbar and during the measurements 5 × 10^− 5^ mbar.

The structure of the formed tribofilms was analyzed by transmission electron microscopy (TEM, FEI TECNAI F20). Energy dispersive X-ray spectroscopy (EDS) was performed within the TEM using an EDAX-AMETEK Apollo XLTW SDD system. For TEM, a thin lamella was prepared using a ThermoFisher Scios II Focused Ion Beam (FIB). The lamella measured approximately 20 × 10 μm² with a thickness less than 100 nm thick in the regions of interest. A protective layer of tungsten is deposited to protect the coating during the cutting process. Phase analysis was performed using Selected Area Electron Diffraction (SAED) patterns. In addition, high-resolution TEM (HR-TEM) imaging was used for phase analysis using lattice spacing measurements from the HR-TEM images.

## Results and discussion

### Structure and chemical characterization of the used MXenes

**Fig. 1:**
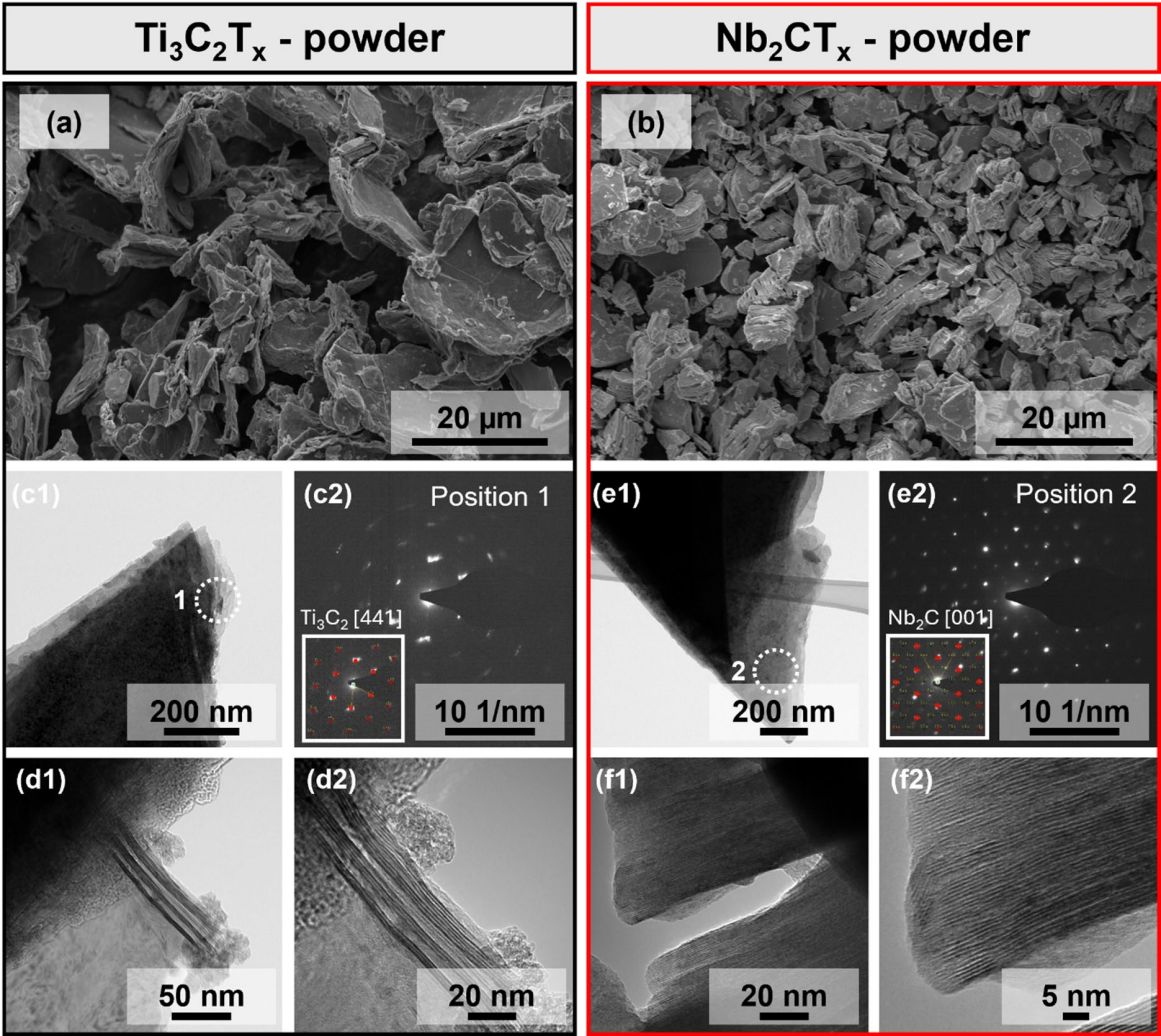
SEM and TEM images of the as-synthesized MXenes Ti_3_C_2_T_x_ and Nb_2_CT_x_. (**a**) SEM image of Ti_3_C_2_T_x_ flakes and (**b**) corresponding morphology of Nb_2_CT_x_. (c1) TEM image of a Ti_3_C_2_T_x_ and the corresponding SAED pattern from position 1 in (c2). (d1) High-resolution TEM bright field imaging showing the layered structure of Ti_3_C_2_T_x_, with increased magnification (d2), providing an interlayer spacing of 1.28 nm. (e1) and (e2) show TEM and SAED images of Nb_2_CT_x_. (f1) and (f2) show a different region of Nb_2_CT_x_ illustrating the layered structure.

The Ti₃C_2_T_x_ used in this study was synthesized by in situ HF etching of Ti_3_AlC_2_, while Nb₂CT_X_ was obtained from Nb_2_AlC_2_. Figure 1 (a) and (b) show the multi-layered flake structures imaged by scanning electron microscopy (SEM). Further details on the layered structure were revealed by transmission electron microscopy (TEM). Figure 1 (c1) shows a single flake of the TEM image of a Ti₃C_2_T_x_ revealing a diffraction distance in the z-direction of 1.04 ± 0.29 nm and of 0.92 ± 0.37 nm for Nb₂CT_X_, (Figure S1, Supporting Information), which corresponds to the values reported in literature^53^. A selected area diffraction pattern was recorded of position 1 Fig. 1 (c2)) confirms a crystalline structure, which could be identified as Ti_3_C_2_ in zone axis (ZA) [441]. Figure 1 (d1) and a larger magnification in (d2) shows the layered structure of the material with interlayer spacing of 1.28 nm (Figure S1 (c), Supporting Information). A TEM image of a Nb₂CT_X_ flake is presented Fig. 1 (e1), with corresponding SAED pattern of position 2 (Fig. 1 (e2), confirming the crystalline structure of Nb₂C in ZA [001]. High resolution TEM images of the structure are shown in (f1) and enlarged (f2).

The phase purity and structural characteristics of the synthesized MXene powder were confirmed by powder X-ray diffraction (Figure S2 (a), Supporting Information). The diffraction pattern displayed a sharp and intense (002) peak at low angles, characteristic of well-ordered MXene layers with increased interlayer spacing, indicating successful etching and delamination of the MAX phase. The absence of residual MAX phase reflections further confirms the high purity of the final product.

Figure S2 (b), Supporting Information illustrates the Raman spectra for two MXenes: Ti₃C_2_T_x_ (black) and Nb₂CT_X_ (red). The Ti-MXenes show typical peak at 207 cm^− 1^ corresponding to an out of the plane Ti-C vibration A_1g_. Peak at 374 cm^− 1^ indicates vibrations coming from the surface terminal groups of MXene, while peak at 626 cm^− 1^ indicates carbon vibrations. Two peaks D and G, coming from carbon vibrations are located at around 1380 and 1546 cm^− 1^. Small peak at 160 cm^− 1^ indicates Ti-O bond vibration^22^.

The Nb-MXene Raman spectrum shows peaks which are in good agreement with those reported in literature. The peaks at 133 and 267 cm^− 1^ represent in-plane and out-of-plane Nb vibrations (Nb–O), respectively. Peaks detected at 251, 435 and 639 cm^− 1^ represent OH, F and O surface termination vibrations, respectively. D and G carbon peaks are present at around 1363 and 1600 cm^− 1^^54^.

### Frictional performance of MXenes

**Fig. 2:**
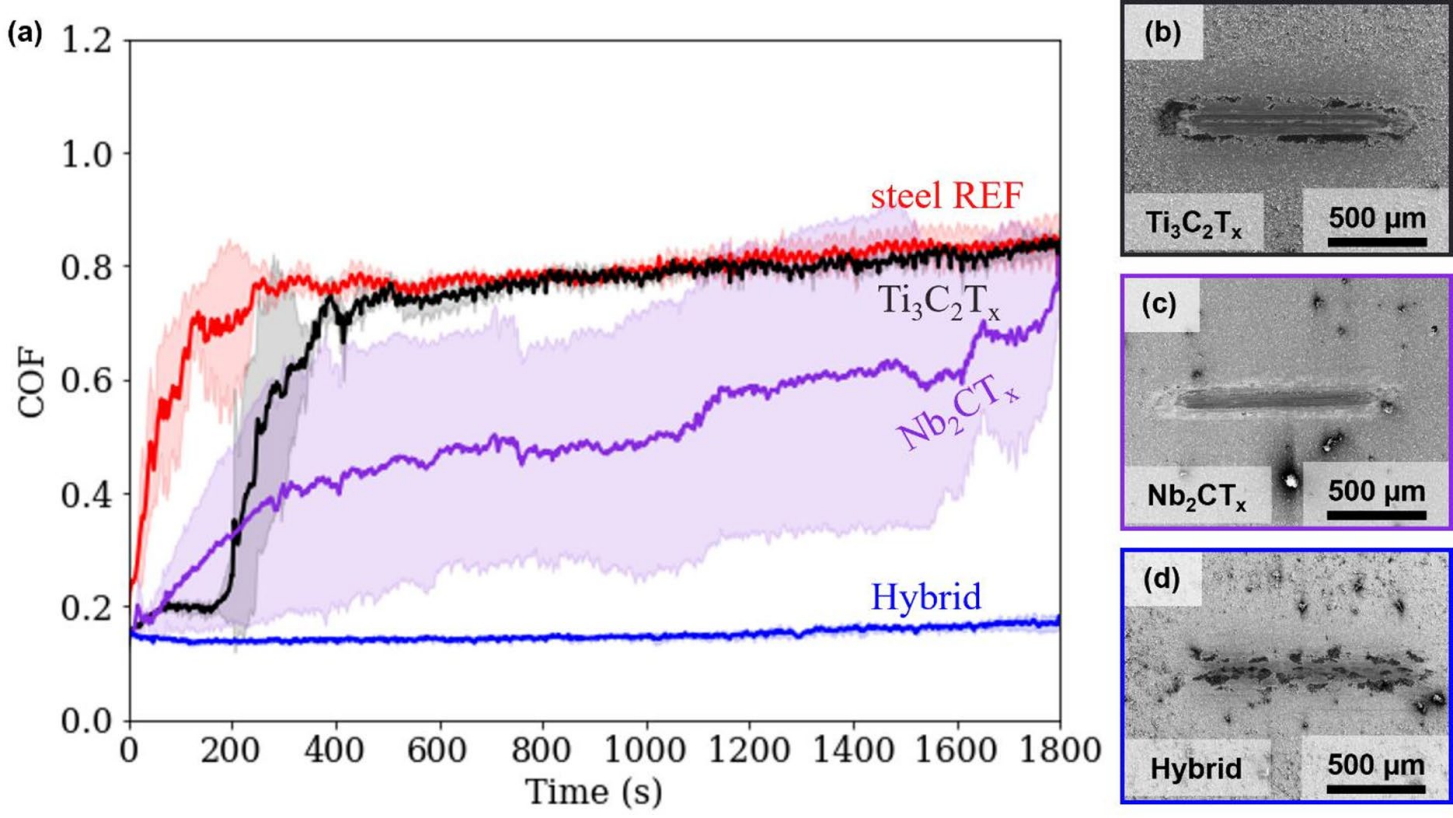
Time-dependent behaviour of the coefficients of friction (**a**) for all MXene coated steel samples against an Al_2_O_3_ counterbody. The average values of three replicates are represented by the solid line, with the corresponding standard deviations indicated by the shaded area. The corresponding wear tracks are shown in for (**b**) Ti_3_C_2_T_x_, (**c**) Nb_2_CT_x_, and (d) the hybrid coating.

The tribological performance of the different MXene coated samples was evaluated using a ball-on-disk tribometer operating in linear sliding mode. Coefficient of friction (COF) values were recorded for Ti₃C_2_T_x_, Nb₂CT_X_ and their hybrid coating in contact with a Al_2_O_3_ counterbody, as illustrated in Fig. 2 (a). Their frictional behaviour is compared to that of an uncoated steel reference sample, tested without any lubrication.

In the case of the uncoated steel sample against the Al_2_O_3_ counterbody, the typical behaviour under dry sliding conditions with a ceramic counterpart is observed, with noticeable fluctuations during the running-in phase and a rapid increase in the COF, regardless of the test conditions^55^. More specifically, the initial COF for the steel reference (steel REF), is approximately 0.25, which quickly increases to almost 0.7 within the first 400 s, after which a steady state value of around 0.8 is reached. The observed friction behaviour corresponds to the typical running-in phase of dry, uncoated metal-ceramic contact under ambient conditions. The significant increase and variability in the COF are primarily attributed to the progressive removal of microscopic surface roughness caused by abrasion and shear at the contact interface^56^. Additionally, ongoing mechanical wear and material transformation generate wear debris, which further contributes to fluctuations and variations in the COF^57^.

In contrast, the Ti₃C_2_T_x_ coated sample held the COF to 0.2 in the first 200 s, to then rapidly diverge to 0.8. The evolution in time of the COF for the Nb₂CT_X_ coating reveals a different trend. The COF started at 0.17, followed by a gradual increase to around 0.5, where it remained stable until around 1200 s before further rising to 0.8. Compared to the reference and Ti₃C_2_T_x_, the Nb₂CT_X_ coating showed a larger standard deviation, indicating greater variability in friction behaviour.

A possible interpretation of the COF vs. time curve suggests that under the applied tribological conditions, the contact pressure of 0.48 GPa led to the removal of the MXene coatings from the contact zone, resulting in material accumulation at the turning points of the wear track. This detachment facilitated the formation of debris and oxides, which acted as abrasives and contributed to the observed increase in COF.

A significant reduction and stability in time of the COF was observed for the hybrid coating. Starting at 0.14, the friction reduction remains consistent and stable throughout the entire test duration, without noticeable fluctuations. By the end of the test, the COF stabilizes at 0.17, demonstrating that the hybrid coating retains its beneficial friction-reducing effects. The results surpass the previous tests, with the hybrid coating achieving 82% reduction in COF with respect to the references.

Panels (b), (c), and (d) of Fig. 2 present the wear track morphologies after the tribological tests for Ti₃C_2_T_x_, Nb₂CT_X_ and the hybrid coating respectively. The wear track morphology of the hybrid coating differs significantly from those of the individual MXene coatings. For the hybrid coating (Fig. 2 (d)), a tribofilm formed by dark patchy traces is observed. In contrast, the wear tracks of the individual MXene coatings (Fig. 2 (b) and (c)) show an uneven film formation, particularly at the track edges and reversal points. Surface analytics is required to clarify the underlying tribological mechanism. To this goal, we carried out various post-test analyses of the substrate surfaces after the friction test. To gain insight into the chemical and structural properties, the results of XPS, Raman, and TEM as well as TEM-EDS analysis are presented in the following sections. Since the most promising behaviour was observed on the hybrid coating, our analysis focuses primarily on this coating.

### Surface analysis of the wear tracks

SEM EDS analysis was carried out on different regions of the wear tracks. For the Ti₃C_2_T_x_ coated sample, no detectable Ti was found in the central region of the wear track (area 4, Figure S4 (a1), Supporting Information), whereas a small amount of detectable Nb was identified in the corresponding region of the Nb₂CT_X_ coated sample (area 4, Figure S4 (b1), Supporting Information). These results suggest that both Ti₃C_2_T_x_ and Nb₂CT_X_ were pushed away from the contact zone and accumulated at the edges, indicating partial coating failure due to lack of adhesion.

**Fig. 3:**
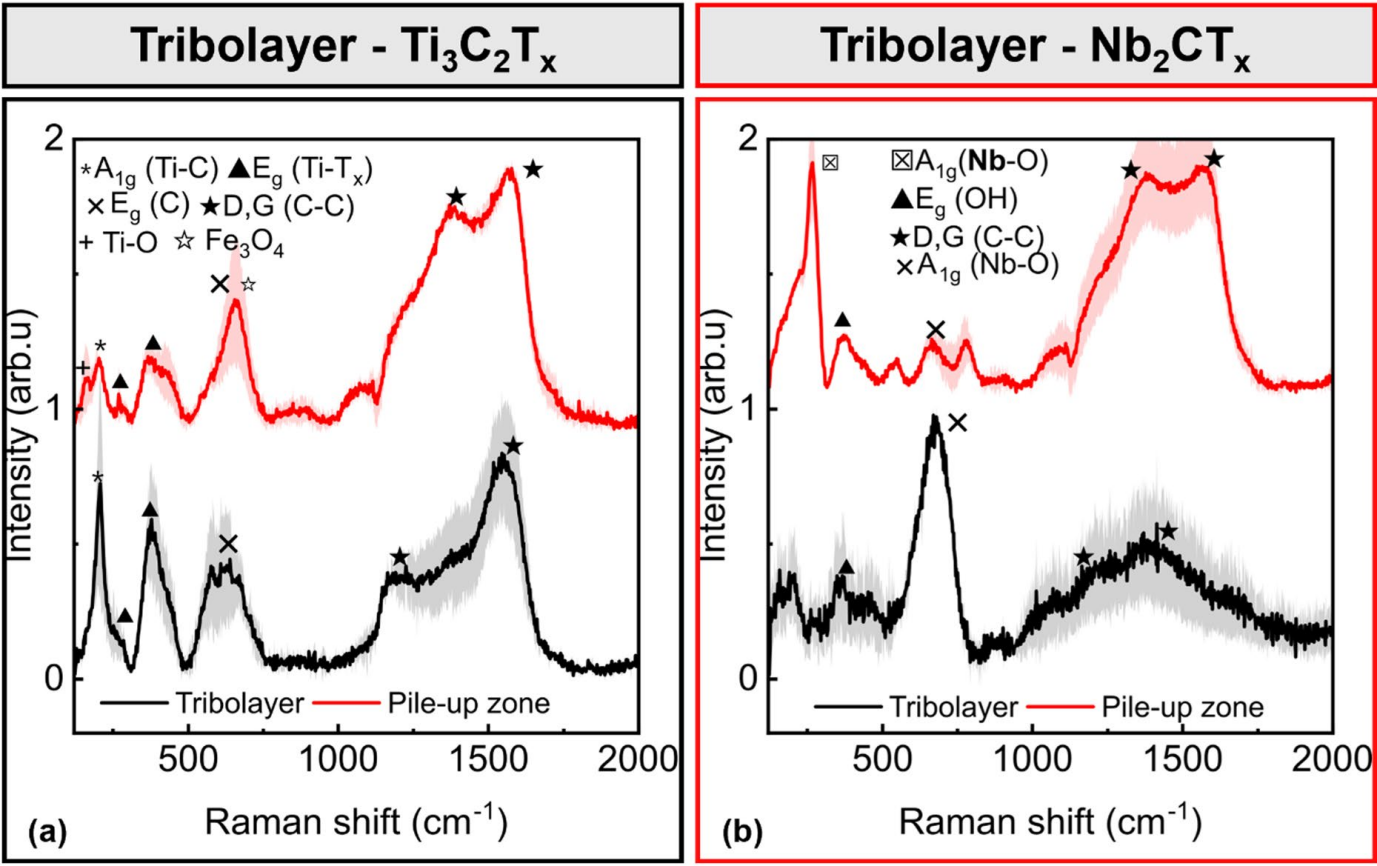
Average Raman spectra measured on wear tracks after tribological tests using different MXenes (*n* ≥ 2): (**a**) Ti_3_C_2_T_x_, (**b**) Nb_2_CT_x_.

We applied Raman spectroscopy to characterize the tribofilms found in the obtained wear tracks (see Fig. 3). The spectra exhibit vibrational modes corresponding to characteristic peaks of MXene powders (see Figure S2 (b), Supporting Information), indicating the chemical stability of the formed tribolayer. In contrast, the pile-up zone reveals a weak Ti-O vibrational mode, which suggests that the MXene is partially oxidised. Additionally, the D and G bands exhibit significantly higher intensity in the pile-up region than the Ti out of-the-plane vibration, indicating an increased carbonaceous contribution. The sharp peak at 659 cm^− 1^ may be due to intensified carbon vibrations or potential contributions from Fe_3_O_4_^58^; however, the overlap of these two signals makes it difficult to identify the individual contributions precisely.

The Raman spectrum of Nb-MXene shows the same characteristic peaks as the powder sample, though with altered intensity ratios (Figure S2 (b), Supporting Information). A pronounced peak at 675 cm^− 1^ is observed in the tribolayer region, which corresponds to Nb-O bond vibrations. The peak intensity is more dominant than the one found in Ti₃C_2_T_x_, suggesting that Nb-based MXene is more prone to oxidation with respect to the Ti counterpart. The results suggest that the tribological test has induced partial chemical instability of the Nb-MXene. In contrast, the pile-up zone exhibits spectral features consistent with those of the original powder, possibly indicating that the MXene flakes that remain in the contact area undergo harsh tribochemical processes, while those able to slide toward the pile-up zone can maintain a less oxidised state.

**Fig. 4:**
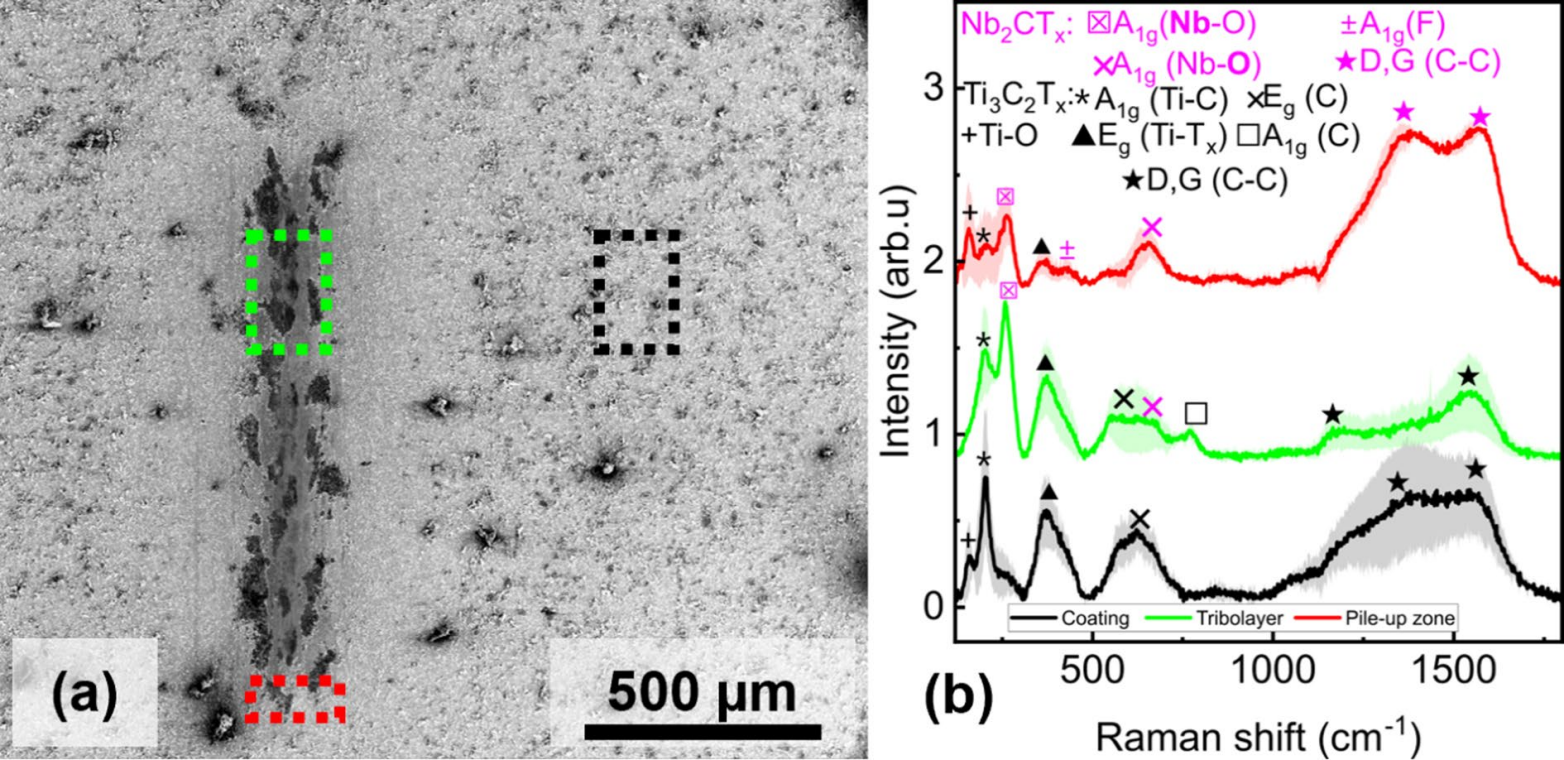
(**a**) SEM micrograph of the substrates wear scar after tribological tests on Hybrid MXene coating, (**b**) Average Raman spectra measured on counterbody areas identified by SEM (same colour code): *n* ≥ 3, n = number of tests.

Finally, Fig. 4 shows the Raman spectra of the hybrid coating evaluated in three points on the surface. The tribolayer (red), the pile-up zone (green), and the untested coating (black). Only Ti-based MXenes were detected in the reference zone of the hybrid coating, with no peaks corresponding to Nb-MXenes vibrations. Notably, no peaks indicating oxidation of MXene were found, suggesting good oxidation stability of the hybrid coating.

The tribolayer in the hybrid MXenes indicates the presence of both types of MXene due to distinct peaks appearing at 203 and 258 cm^− 1^, representing Ti and Nb vibrations, respectively.

 The relatively low intensity of the D and G bands indicates a reduced degree of carbon disorder within the tribolayer. Additionally, a new vibrational mode observed at 770 cm⁻¹, attributed to the A₁g(C) phonon, emerges in the Raman spectrum. Notably, this feature is absent in both the pristine MXene powders (see Figure S2 (b), Supporting Information) and the singly MXene-coated samples (Figure S3 for Light-microscope images and Table S1 for details on measured surface roughness, Supporting Information), which exhibit pronounced carbon-related vibrations but lack the 770 cm⁻¹ peak. This distinction suggests that the Raman signature reflects a synergistic effect arising from the hybrid coating, surpassing the characteristics of the single-coated configurations.

Specifically, the diminished D and G band contributions, coupled with the appearance of the A₁g(C) mode, imply a surface restructuring within the tribolayer. This restructuring likely results in better ordering or exposure of MXene flakes. It is proposed that the kinetic energy imparted during tribological stress facilitates this transformation by reducing carbon disorder and promoting the alignment of MXene flakes at the interface with the counterbody. Analysis of the pile-up zone reveals a weak Nb-O peak, which is also present in the reference powder. This suggests that partially oxidised Nb-MXenes are being ejected from the contact zone into the pile-up zone by the oscillatory motion of the counterbody, while unoxidised particles form a stable tribological film.

 X-ray photoelectron spectroscopy (XPS) was conducted on both the as-deposited coating (reference) and the wear track (tribolayer) to further examine the oxidation state of niobium. Specifically, we analysed the C 1 s, F 1 s, Nb 3 d, O 1 s, and Ti 2p core levels (see Figure S10, Supporting Information). The tribolayer exhibited a higher concentration of oxides, particularly TiO₂ and Nb₂O₅, compared to the reference surface. Quantitative analysis revealed that the oxide-to-carbide ratio for Nb 3 d exceeded unity in both the reference and tribolayer regions, whereas for Ti 2p, this ratio remained below one (see Table S3, Supporting Information).

These findings suggest that Nb-based MXenes are more susceptible to oxidation under tribological stress, while Ti-based MXenes retain a more carbide-like character. This trend is consistent with the Raman spectroscopy results, which also indicated a higher degree of structural preservation in the Ti-based components.

### Transmission electron microscopy (TEM) analysis

To gain deeper insight into the structure and composition of the tribofilms formed in the MXene hybrid coating, transmission electron microscopy (TEM) was employed to examine the wear track. A focused ion beam (FIB) was used to extract a cross-sectional TEM lamella from the edge region of the hybrid coating, approximately at the centre of the wear track (see Figure S5, Supporting Information). The cross-sectional TEM image reveals a compact, flat tribofilm with an average thickness of approximately 230.5 ± 82 nm following tribological testing (Figure S6 (h)-(i), Supporting Information).

**Fig. 5:**
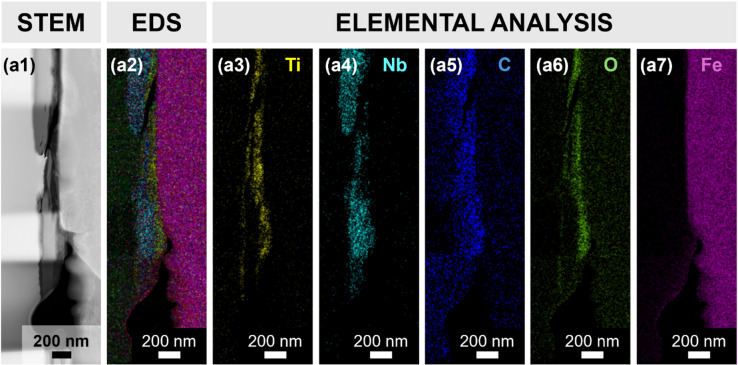
Scanning transmission electron microscopy (STEM)-EDS analysis of the wear track for the hybrid coated sample. (a1) shows the STEM HAADF image and (a2) the corresponding EDS elemental overlay image, respectively, with colour-coded distributions. Elemental maps are shown individually for (a3) titanium, (a4) niobium, (a5) carbon, (a6) oxygen and (a7) iron. (A localized dark green signal in the left region of (a2) corresponds to tungsten (W), which was applied as a protective layer during FIB sample preparation.).

To further investigate the elemental distribution within the tribolayer and assess compositional changes induced by sliding, energy-dispersive X-ray spectroscopy (EDS) mapping was performed. The elemental maps show a relatively uniform distribution of Ti and Nb throughout the tribofilm (Fig. 5 (a3) and Fig. 5 (a4)). Notably, the oxygen signal (Fig. 5 (a6)) closely follows the Ti distribution, consistent with the presence of oxygen-containing surface terminations on Ti-based MXenes.

Interestingly, the cross-sectional analysis reveals a stratified structure within the tribofilm: Ti-rich regions are predominantly located near the substrate, while Nb-rich regions are concentrated toward the top surface. This stratification does not correspond to the original sequential deposition of the coating layers, suggesting that it results from tribologically induced redistribution during sliding.

**Fig. 6:**
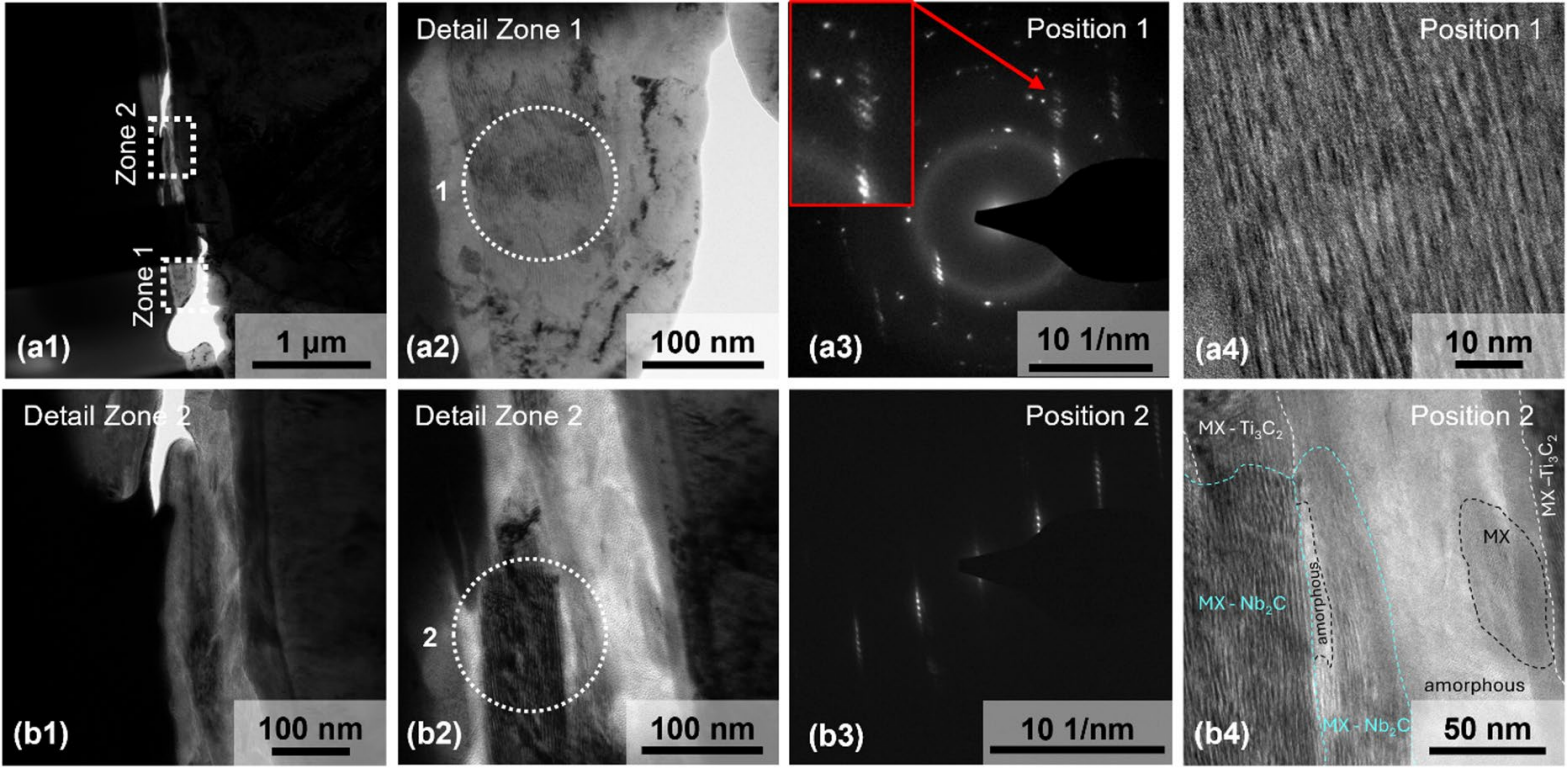
TEM analysis of the wear track of the hybrid coating. (a1) shows a TEM overview image of the lamella with marked regions Zone 1 and Zone 2. For more detailed analysis, (a2) presents a bright field TEM image of the tribofilm in Zone 1, while (a3) displays the corresponding selected area electron diffraction (SAED) pattern at position 1, confirming nanocrystallinity. A high-resolution TEM image in (a4) further reveals the layered structure at position 1. (b1) displays a magnified overview of Zone 2, while (b2) shows a higher-magnification TEM image of this zone. The corresponding SAED pattern of position 2 is shown in (b3) and confirms the single crystallinity of the Ti_3_C_2_-MXenes, which is also discussed in more detail in the Supportive Information in Figure S7. The high-resolution TEM image in (b4) provides further insights into the nanoscale layering and its arrangement within the tribofilm at this location.

TEM was used to examine the microstructure of two distinct regions within the tribofilm (Zone 1 and Zone 2), and selected area electron diffraction (SAED) patterns were acquired for the evaluation and determination of the crystal phase after the deformation caused by the tribo-tests see Fig. 6).

In Zone 1, TEM images (Fig. 6 (a2)) reveal that the tribofilm possesses a mixed microstructure. A TEM image with a lager magnification taken at position 1 highlights the layered structure of the MXene flakes (Fig. 6 (a4)). Figure 6 (a3) confirms nanocrystallinity, indicated by the formation from spots to rings. Further, the diffraction patterns show a spread in the radio of the diffraction spots, which is due to a slight but continuously change in the crystal lattice parameters.

In contrast, the pre-tested MXene flakes shown in Fig. 1 (c2) and (e2) indicate a pure single crystal structure. Thus, it can be concluded that this change in the crystal structure is caused by the tribological tests.

In Zone 1, an amorphous ring is clearly visible in the diffraction pattern. It is stemming from the left and right outer part in the selected area diffraction aperture – see Fig. 6 (a2). The amorphous regions indicate a complete destruction of the crystallinity of the Nb and Ti flakes, caused by the tribological tests. The diffraction pattern shown in Fig. 6 (b3) shows the crystalline structure of Ti₃C_2_T_x_. More details are available in the Supporting Information (Figure S7 and Figure S8).

Moreover, Fig. 6 (b4), shows the area of Zone 2 at higher magnification in a high-resolution TEM image, where crystalline and amorphous regions can be identified immediately.

For Nb₂CT_X_ and Ti₃C_2_T_x_ MXenes (crystal structure confirmed by mean lattice spacing distances) an area named “MX” is defined, where MXene-layers are visible but not clearly identifiable. Moreover, an amorphous region runs through the centre of the picture. Details on the calculated lattice distances are reported in Figure S9 (Supporting Information). It could not be observed any gap between the different areas, suggesting that the MXene-flakes are compacted together in different configurations after the tribological tests. The diffraction patterns could be clearly identified as the phases Nb₂C and Ti₃C₂, confirming that a part of the tribofilm maintains the initial crystallinity of the unencumbered flakes.

### Tribological mechanism of Nb-Ti MXene hybrid coating

The developed hybrid coating shows exceptional COF performances (Fig. 2), and the surface analytics confirm the presence of both MXenes types in the wear tracks (Figs. 4 and 5), where they present diverse crystallographic structures (Fig. 6). Interestingly, the cross-section investigation by TEM-EDS presents a different arrangement of the MXene flakes with respect to the initial application sequence: the hybrid coating is prepared by depositing first the Nb₂CT_X_ solution followed by the Ti₃C_2_T_x_ solution, creating a mixture of the two materials which could not be confirmed after the tribological test.

Hereby we propose an adaptive reconfiguration as a possible tribological mechanism. Upon the onset of tribological stress, the system self-organizes into a configuration that minimizes friction and wear.

It is worth noting that this mechanism activates to mitigate the coating peeling off, optimizing the bonding adhesion of the solid lubricant onto the counterbody, ultimately redefining the sliding plane. The hybrid MXene solid lubricant coating does not behave as an isotropic film, but rather as a discrete system with nanoscale materials. Typically, solid lubricants made from a single material move along the sliding plane following its direction. The tribological mechanism in this case are discussed as reservoir mechanism and agree with isotropic coating lubrication behaviour^18,59^. In contrast, the hybrid solution allows an additional degree of freedom as the two types of MXenes are mobile on the direction orthogonal to the sliding plane as well (see cross-sectional TEM, Figs. 5 and 6). This mobility and re-organization ability is key to understand the value of hybrid solutions over one-type solid lubricant coating solutions.

We suggest the self-organization is due to the following aspect: Ti₃C_2_T_x_, which has a better chemical affinity to the substrate with respect to Nb₂CT_X_ due to the stable Ti-Fe interfacial bonding, is displaced at the substrate interface, forming a stable and adherent layer. Consequently, the sliding interface transitions to one dominated by Nb₂CT_X_ vs. Ti₃C_2_T_x_ flake interactions, which lowers the COF due to interlayer shearing between the MXenes species.

Notably, Raman spectroscopy indicates that the pile-up zone is predominantly composed of oxidised Nb₂CT_X_, confirming that in this adaptive configuration, Nb-based MXenes migrate to the counterbody interface and are subsequently transported out of the contact zone. However, due to the poor chemical stability of Nb₂CT_X_, it collapses to its oxidised form as showed by XPS. Both TEM and Raman spectroscopy highlight the significant role of carbon: Raman reveals distinct differences in carbon vibrational modes between the wear track and the pile-up zone (Fig. 4), while TEM shows amorphous regions (Fig. 6) and TEM-EDS a broad distribution of carbon within the wear track (Fig. 5). Additionally, XPS analysis indicates that Ti₃C_2_T_x_ retains its carbide nature even after tribological stress, rather than converting to an oxide.

In summary, under tribological stress, the hybrid coating undergoes a dynamic reorganization that leverages the distinct properties of its components. The Nb₂CT_X_ phase, owing to its high tribochemical reactivity, contributes to the formation of an initial sacrificial surface layer. Meanwhile, the Ti₃C_2_T_x_ phase, thanks to the strong mechanical stability and the better chemical affinity to the substrate, sustains a low COF by forming a robust layer on which Nb₂CT_X_ can slide. Importantly, Nb₂CT_X_ is not entirely consumed in the sacrificial process; it remains active within the wear track and continues to facilitate sliding in conjunction with Ti₃C_2_T_x_. This stress-induced redistribution results in a synergistic interaction between the two MXenes, enabling superior tribological performance that neither material achieves on its own, as demonstrated in Fig. 2.

## Conclusion

This study presented a systematic investigation into the tribological behaviour of MXene hybrid coatings (Nb₂CT_X_ and Ti₃C_2_T_x_) under dry sliding conditions. Through a multi-technique characterization approach including SEM-EDS, Raman spectroscopy, XPS, and TEM, we demonstrate that the hybrid MXene coating significantly outperforms the individual components in terms of friction reduction and wear resistance.

 The hybrid coating exhibits a stable and low coefficient of friction (COF < 0.2), attributed to the formation of a compact, patchy tribofilm. The low and stable COF found at the selected contact pressure (0.48 GPa) makes the hybrid coating competitive in the framework of different solid lubricants (see the list in Table S2). These performances are attributed to the tribofilm forming from a stress-induced adaptive reconfiguration, wherein Ti₃C_2_T_x_ preferentially anchors to the substrate due to its superior interfacial bonding, while Nb₂CT_X_ migrates toward the sliding interface, acting as a sacrificial layer. This dynamic redistribution results in a stratified tribolayer with enhanced mechanical integrity and tribochemical resilience.

Raman and XPS analyses confirm that within the hybrid tribofilm, Ti₃C_2_T_x_ retains its carbide nature under tribological stress, while Nb₂CT_X_ undergoes partial oxidation, contributing to the formation of a lubricious oxide-rich surface. TEM imaging reveals a nanostructured tribofilm with both crystalline and amorphous domains, further supporting the hypothesis of synergistic interaction between the two MXenes.

It is important to highlight that the adaptive reconfiguration described hereby is a characteristic of the selected hybrid MXene coating and tribological system. Further investigation into various hybrid solid lubricant combinations and tribological pairings is essential to develop a comprehensive understanding of hybrid lubrication strategies. Moreover, numerical simulations encompassing tribological interfaces, environmental conditions, and the chemical characteristics of the solid lubricants—such as the choice of transition metals, carbide or nitride phases, and surface terminations—could significantly accelerate insights into the behaviour and optimization of hybrid MXene-based solid lubricant coatings.

These findings highlight the potential of hybrid MXenes as a powerful strategy for designing next-generation solid lubricants. By leveraging the complementary properties of different MXene species, it is possible to engineer coatings with tailored tribological performance, opening new avenues for advanced applications in energy-efficient and high-performance mechanical systems.

## Supplementary Information

Below is the link to the electronic supplementary material.

Supplementary Material 1

Supplementary Material 2

Supplementary Material 3

## Data Availability

Data will be available upon sensible request.
