# Supplementary material for: Hybrid MXene coatings: unlocking synergistic lubrication properties of Ti3C2Tx and Nb₂CTx MXenes for improved tribological performance
Source: Sci Rep. 2025 Dec 22;15:45111. doi: 10.1038/s41598-025-32533-6 (PMC12749883; doi:10.1038/s41598-025-32533-6)
Supplement: Supplementary file 3 — Supplementary Material 3 [file 41598_2025_32533_MOESM3_ESM.docx]

**Supporting Information**

**Hybrid MXene Coatings: Unlocking Synergistic Lubrication Properties of Ti₃C₂Tₓ and Nb_2_CT_x_ MXenes for Improved Tribological Performance**

Christina Danecker^1^, Sabine Schwarz^2^, Marko Piljevic^3, 4^, Jakob Rath^5^, Martin Nastran^1,6^ Bernhard C. Bayer^6,^ Michael Naguib^7^, Ahmad Majed^7^, Karamullah Eisawi^7^, Pierluigi Bilotto^1*^ and Carsten Gachot^1*^

^1^ Institute of Engineering Design and Product Development, Research Unit Tribology, TU Wien, Lehargasse 6, Building BA, 9th floor 1060, Austria

^2^ University Service Center for Transmission Electron Microscopy (USTEM), TU Wien, Stadionallee 2, 1020, Vienna, Austria

^3^ AC2T research GmbH, Viktor-Kaplan-Straße 2/C, Wiener Neustadt, 2700, Austria

^4^ CEST GmbH, Centre for Electrochemical Surface Technology, A-2700, Wiener Neustadt, Austria

^5^ Analytical Instrumentation Center, TU Wien, Lehargasse 6, 1060, Vienna, Austria

^6^ Institute of Materials Chemistry, TU Wien, Getreidemarkt 9, 1060 Vienna, Austria

^7^ Tulane University, School of Science and Engineering, 208 Stanley Thomas Hall (US)

***Corresponding Author**

E-Mail: [carsten.gachot@tuwien.ac.at](mailto:carsten.gachot@tuwien.ac.at), [pierluigi.bilotto@tuwien.ac.at](mailto:pierluigi.bilotto@tuwien.ac.at)


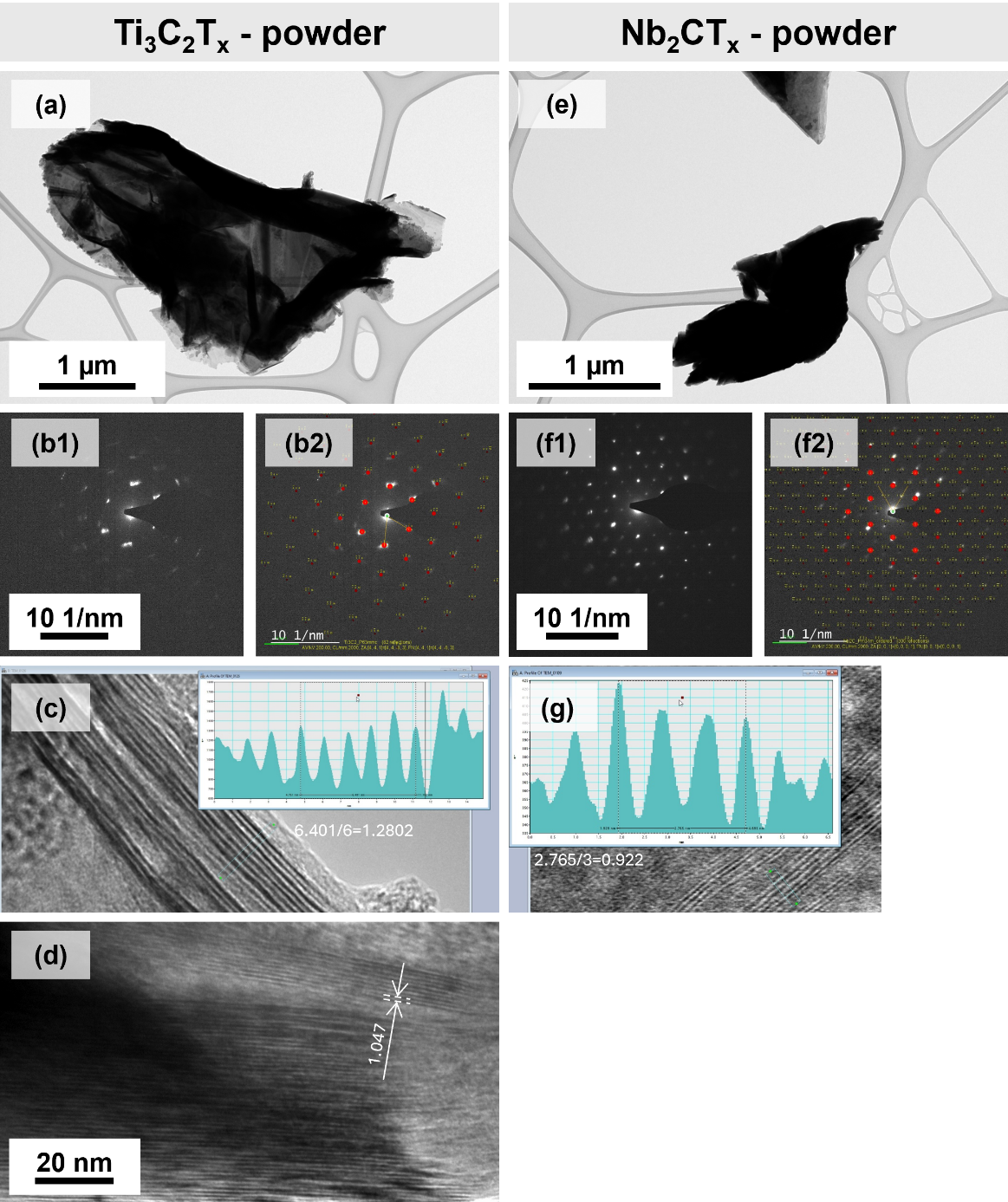


Figure S1: TEM images of the synthesized MXene powders (a) lower magnification TEM image of the as-synthesized few-layer Ti₃C₂Tₓ nanosheet. (b1) SAED pattern; (b2) confirms its crystalline structure and the Ti₃C₂ phase along the [441] zone axis. (c) Measured layer thickness of Ti₃C₂Tₓ is 1.28 nm; (d) an additional measurement from a HRTEM image yields interlayer spacing of 1.04 nm. (e) Corresponding TEM image of a few-layer Nb₂CTₓ nanosheet. (f1) and (f2) verify the crystalline structure and confirm the Nb₂C phase along the [001] zone axis. The corresponding HRTEM image (g) shows a measured interlayer spacing of 0.92 nm for Nb_2_CT_x_.

Powder X-ray diffraction (PXRD) measurements were performed on a Θ/Θ Bragg–Brentano diffractometer equipped with a copper anode (Cu Kα radiation, λ = 1.5406 Å). The instrument was configured with an anode-to-sample distance of 240 mm and a sample-to-detector distance of 240 mm. Data collection was carried out using a sample spinner operated without any specific sample environment. Diffraction patterns were recorded using an X’Celerator semiconductor detector with an active detection range of 2.1°.


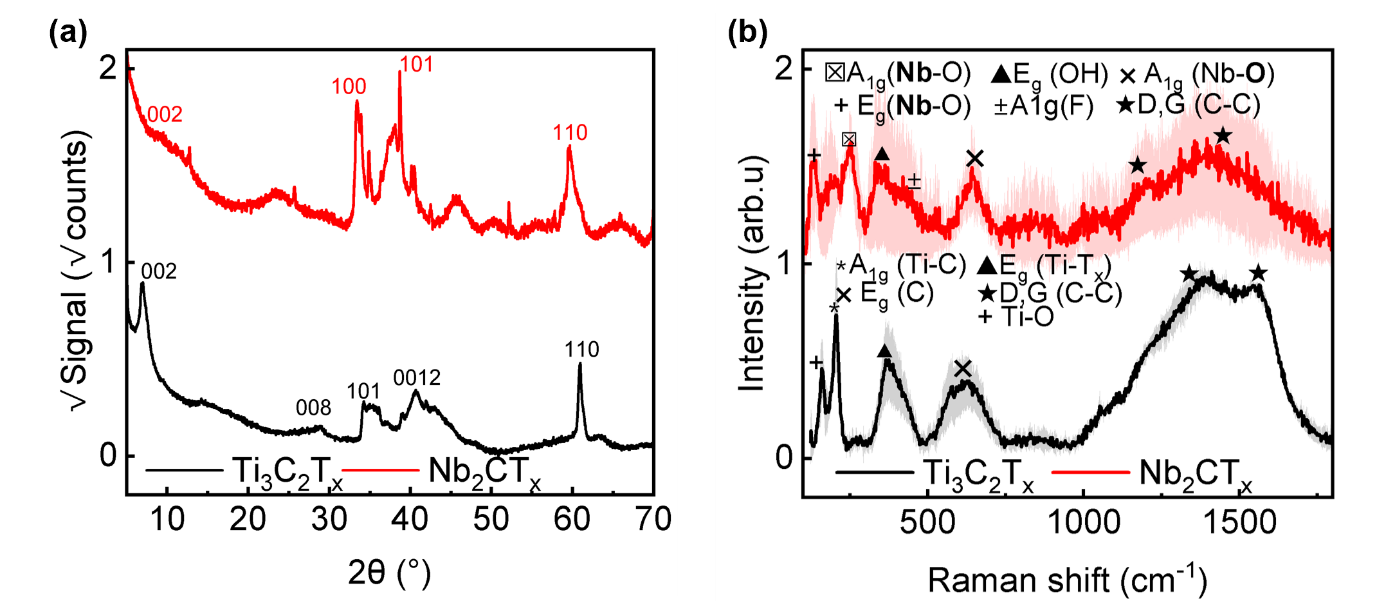


Figure S2: (a) XRD patterns of Ti_3_C_2_T_x_ and Nb_2_CT_x,_ indicating their phase composition and preferred orientations. The indexing was performed by comparing to the state of the art on the MXenes employed for the hybrid coating.[1], [2], [3], [4] (b) Normalized mean Raman spectra of MXene powder with standard deviation (n≥3): Ti_3_C_2_T_x_ (black) and Nb_2_CT_x_ (red).


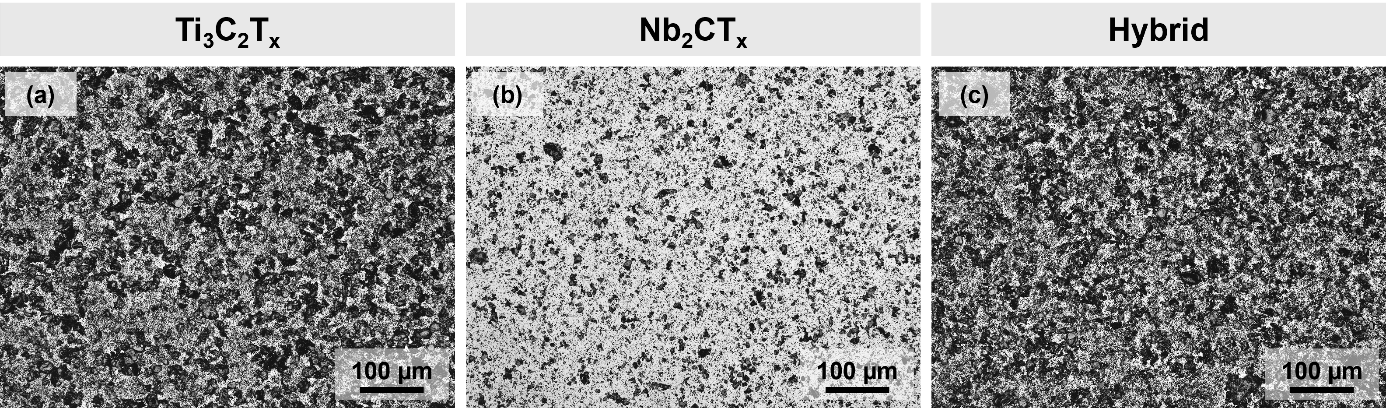


Figure S3: Light-microscope images of the air sprayed coatings for (a) Ti_3_C_2_T_x_ (b) Nb_2_CT_x_ (c) Hybrid

Table S1: The surface roughness of the respective coatings was determined based on light microscopy images equivalent to those shown in Figure S3.

|  | **S_a_**  **_(µm)_** | **S_z_**  **_(µm)_** | **S_q_**  **_(µm)_** | **S_sk_**  **_(µm)_** | **S_ku_**  **_(µm)_** |
| --- | --- | --- | --- | --- | --- |
| **Ti_3_C_2_T_x_** | 1,197 | 1,638 | 26,204 | 2,147 | 10,186 |
| **Nb_2_CT_x_** | 0,168 | 0,273 | 6,776 | 3,354 | 26,039 |
| **Hybrid** | 0,868 | 1,253 | 24,409 | 2,493 | 13,930 |


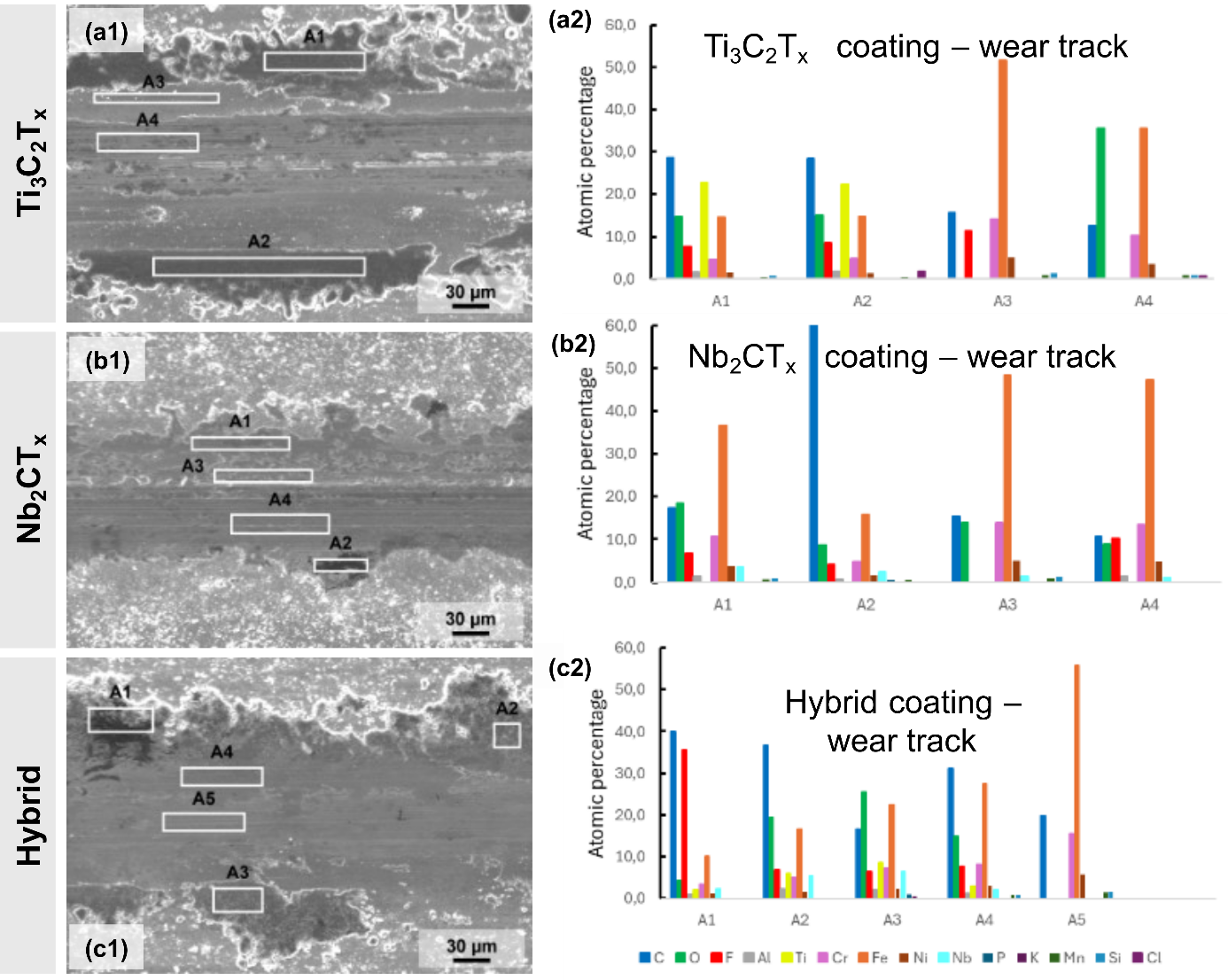


Figure S4: Energy-dispersive X-ray spectroscopy (EDS) analysis of the wear tracks for the different coatings. For the Ti₃C₂Tₓ coating (a1), specific regions within the wear track were selected for analysis, with the corresponding elemental distribution maps shown in (a2). Similarly, the Nb₂CTₓ coating (b1) was examined at marked areas within the wear track, and the resulting elemental maps are presented in (b2). For the hybrid coating (c1), EDS measurements were also conducted at designated regions, with the respective element distribution results illustrated in (c2). These mappings provide insight into the elemental composition and spatial distribution of the coatings after tribological testing.


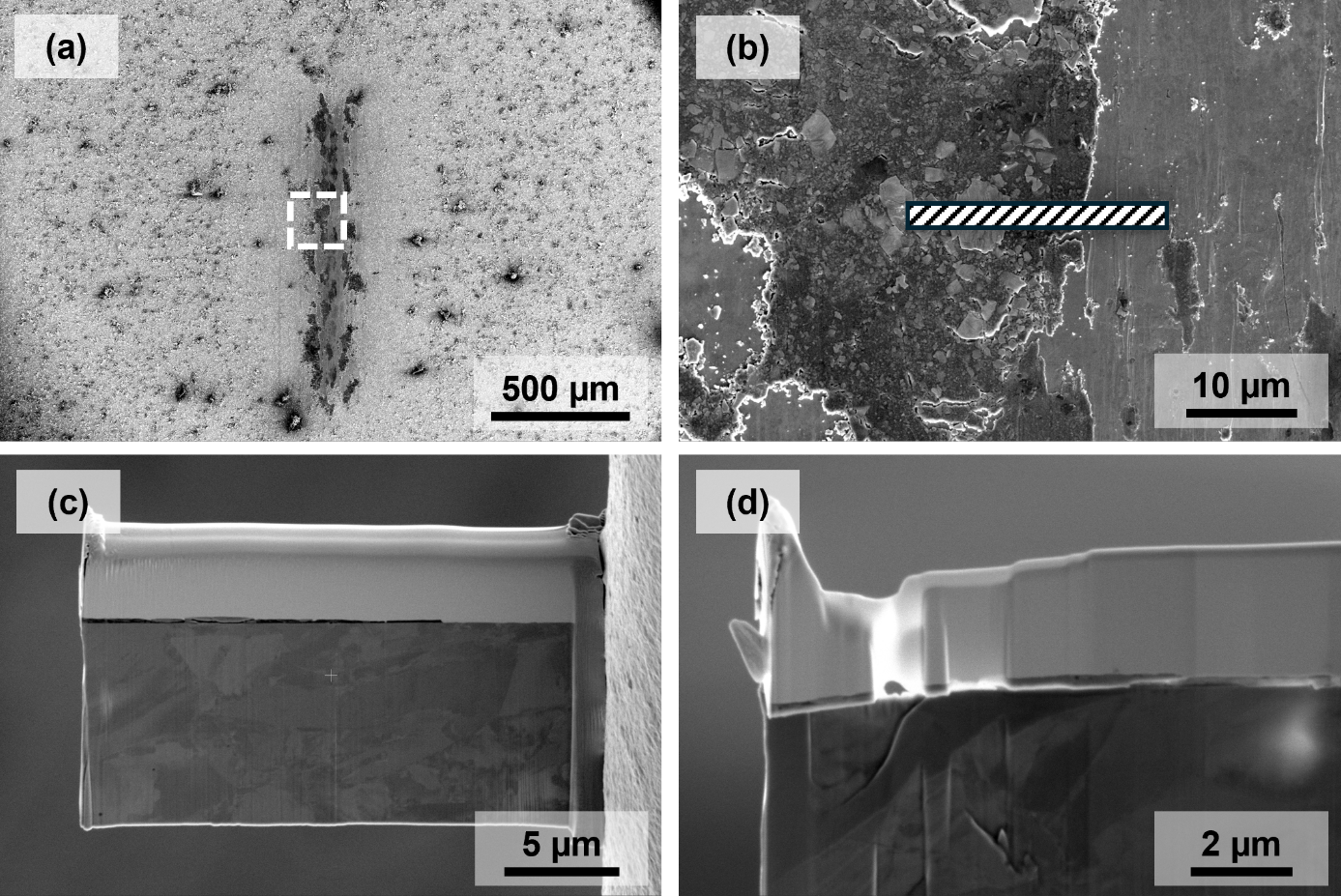


Figure S5: Overview of the TEM lamella preparation from the wear track of the hybrid coating. (a) SEM image of the wear track after a linear sliding friction test. (b) Zoomed-in section highlighting the region of interest showing the FIB cut position. (c) resulting cross-sectional TEM lamella. (d) presents the final TEM lamella, featuring two thinner regions specifically prepared for high-resolution TEM (HR-TEM) analysis.

Table S2: Summary of friction coefficients for different solid lubricants and hybrid coatings as reported in the literature

| **Coating** | **Contact pressure (GPa)** | **Counterbody** | **Speed (mm/s)** | **COF**  **(steady state)** |
| --- | --- | --- | --- | --- |
| **MXene Ti_3_C_2_T_x_** | 0.35 [5], 0.46 [6],  0.3 - 0.5 [7], 0.3 [8],  0.5 - 0.8 [9], 0.48 [this study] | 6 mm Al_2_O_3_ [5],  5 mm Al_2_O_3_ [6]_,_  4 mm AISI 52100 [7], 6 mm Si_3_N_4_ [8],  4 mm tungsten carbide [9], 6 mm Al_2_O_3_ [this study] | 1 [5], 2 [6], 2.5-6 [7], 1 [8],  2.5 [9],  1 [this study] | 0.21 [5], 0.16 [6],  0.2 - 0.3 [7], 0.13 - 0.2 [8], 0.11 - 0.5 [9],  0.17* [this study] |
| **MXene Nb_2_CT_x_** | 0.48 [this study] | 6 mm Al_2_O_3_ [this study | 1 [this study] | 0.17*[this study] |
| **Hybrid: Ti_3_C_2_T_x_ + Nb_2_CT_x_** | 0.48 [this study] | 6 mm Al_2_O_3_ [this study] | 1 [this study] | 0.14 - 0.17 [this study] |
| **Hybrid: Ti_3_C_2_T_x_ + MoS_2_** | 0.5 - 0.8 [9], 0.8 [10] | 4 mm tungsten carbide [9], 10 mm AISI 52100 [10], | 2.5 [9],  5 [10] | 0.11-0.32[9], 0.15 [10] |
| **Hybrid: Ti_3_C_2_T_x_ + Graphene** | 0.006 [11] | 9.526 mm diameter stainless steel ball coated with diamond-like  carbon (DLC) [11] | 100 [11] | 0.0042 [11] (N2 atmosphere) |
| **MoS_2_** | 0.5 - 0.8 [9], 0.45 [12], | 4 mm tungsten carbide [9], 3.2 mm 440C [12], | 2.5 [9],  1 [12] | 0.32 [9],  0.1 [12], |
| * until coating removal | | | | |


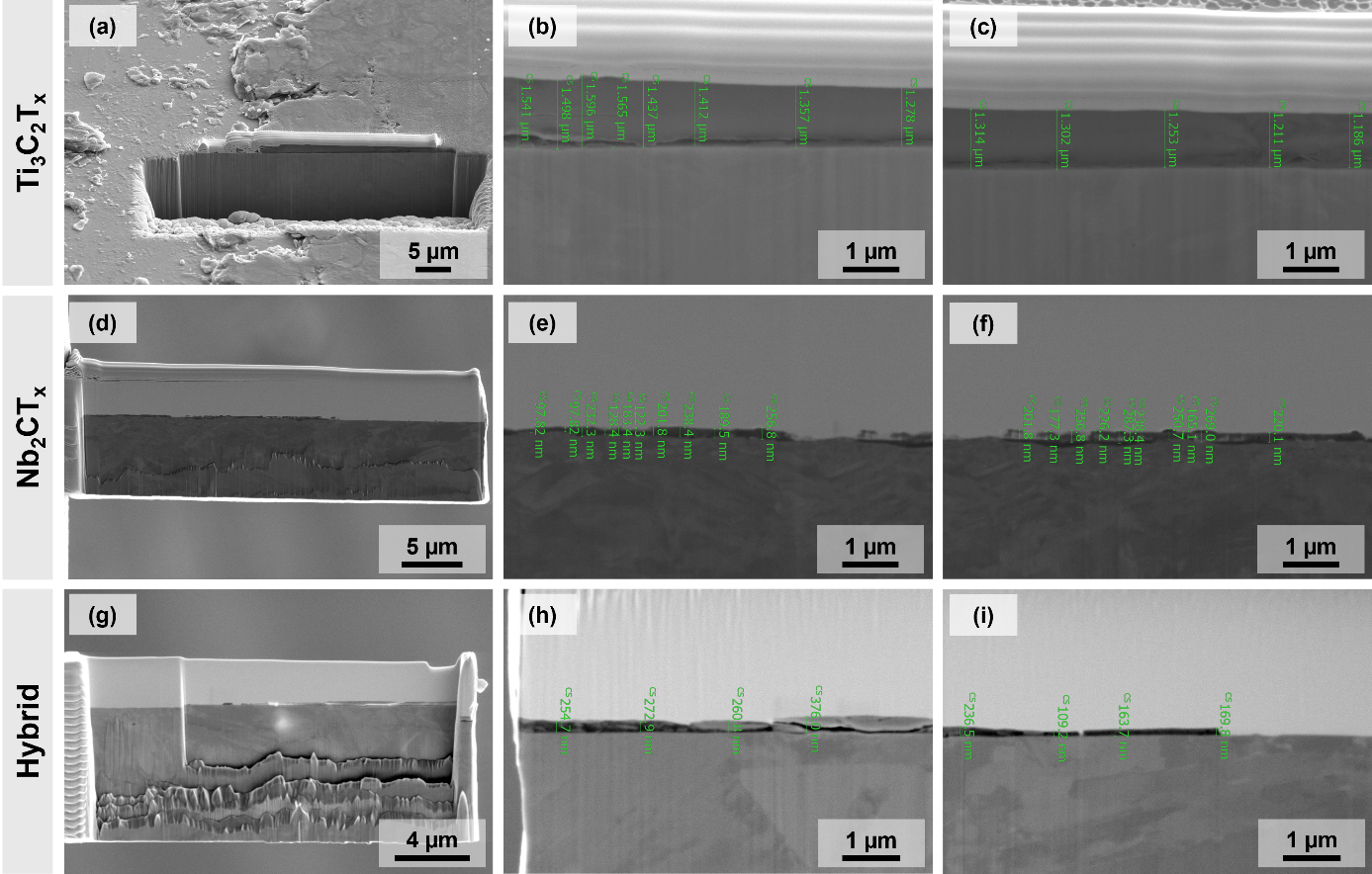


Figure S6: Cross-section and TEM lamellas with measured tribofilm thicknesses of the different coatings: (a) Cross-section of the tribofilm on the Ti_3_C_2_T_x_ coating (b) – (c) corresponding thickness measurement showing an average of 1383 ± 142 nm. (d) TEM lamella from the tribofilm of the Nb_2_CT_x_ coating (e) – (f) measured tribofilm thickness 202 ± 56 nm. (g) TEM lamella extracted from the tribofilm of the hybrid coating (h) – (i) measured tribofilm thickness 230 ± 82 nm.


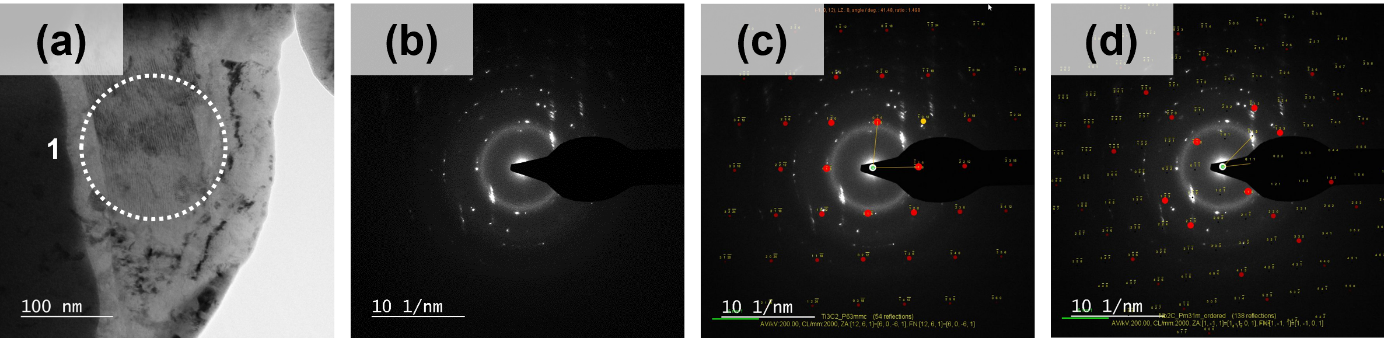


Figure S7: (a) TEM overview image of the lamella from the hybrid coating after the friction test, with a marked area (position 1) selected for detailed analysis. The corresponding selected area electron diffraction (SAED) pattern acquired at this position is shown in (b). (c-d) Further analysis of the diffraction pattern shows an overlap of the two phases: Ti_3_C_2_ in ZA [12,6,1] (c) and Nb_2_C in ZA [1,-1,1] (d).


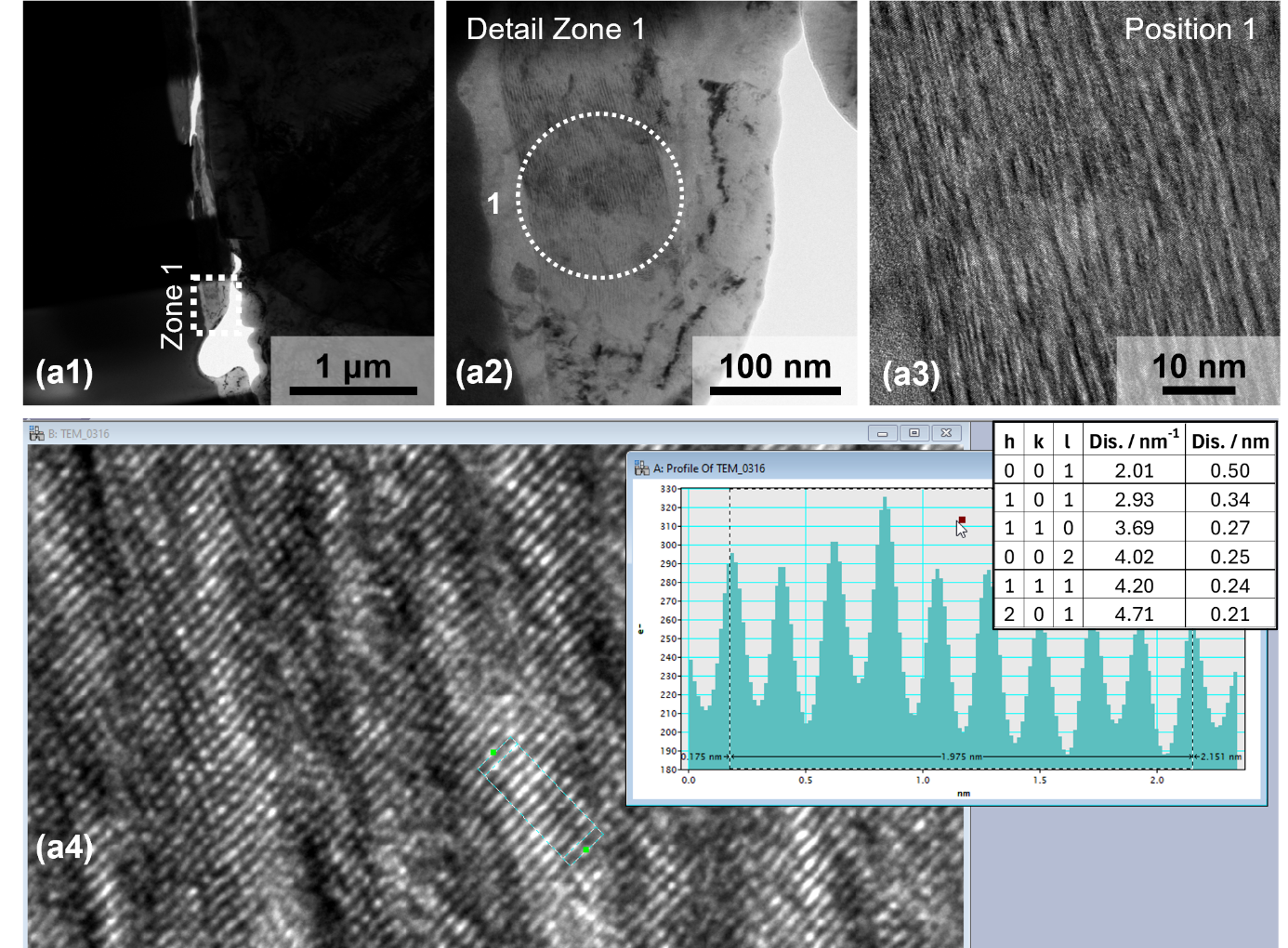


Figure S8: TEM characterization of the wear track of the hybrid coating: (a1) STEM overview image, (a2) bright field TEM image of the tribofilm in Zone 1. (3) HR-TEM image taken at position 1 while (a4) shows the measurement of the lattice spacing.


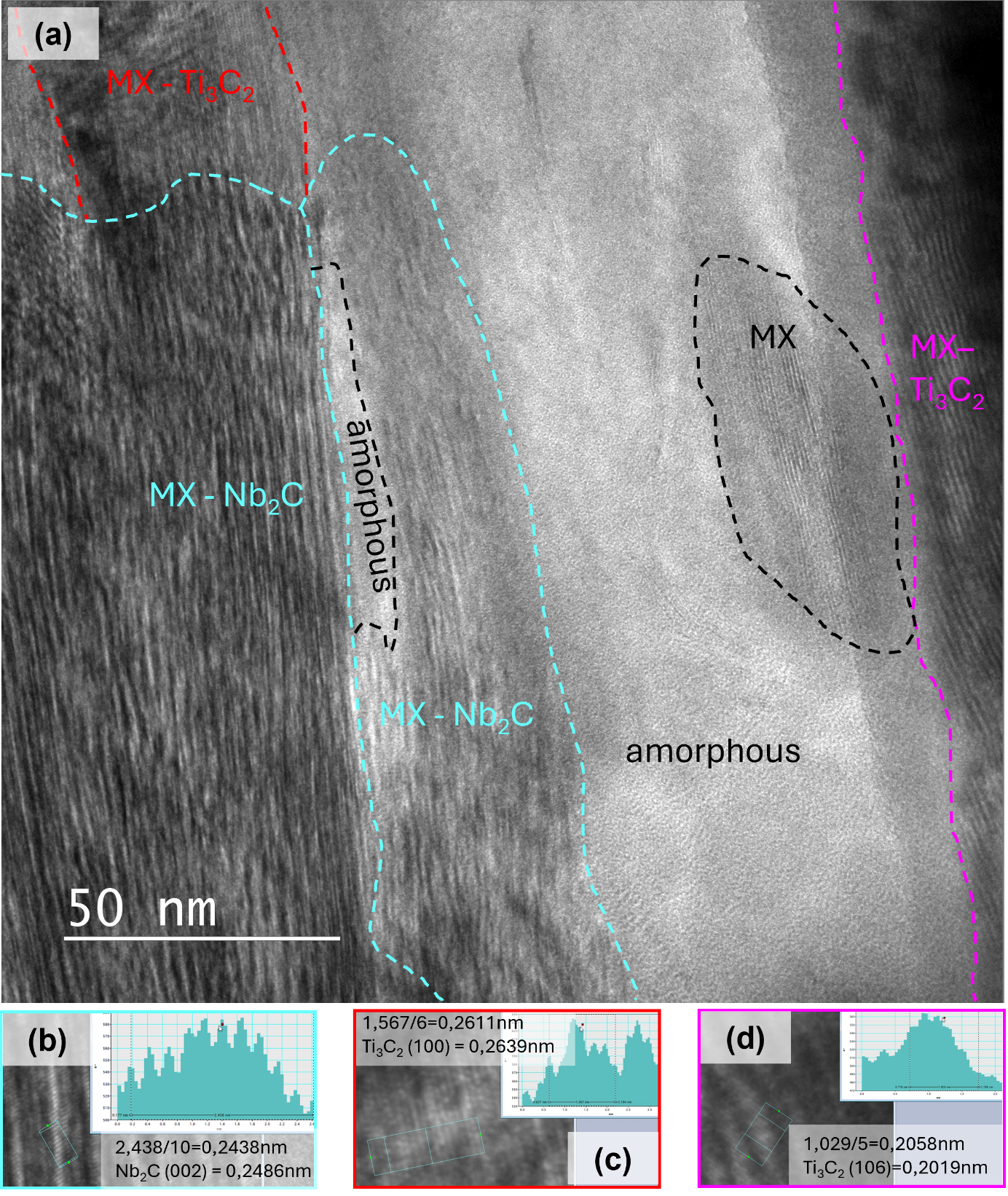


Figure S9: Transmission Electron Microscopy (TEM) analysis of the wear track on the hybrid coating. (a) HR-TEM analysis of the TEM lamella extracted. (b) The lattice plane distance measurement shows a mean lattice space of 0.24nm, which is in good agreement of the Nb_2_C (002) reflex. Same for (c), measured 0,26nm for Ti_3_C_2_ (100) reflex, and (d) 0,21 for Ti_3_C_2_ (106) reflex.


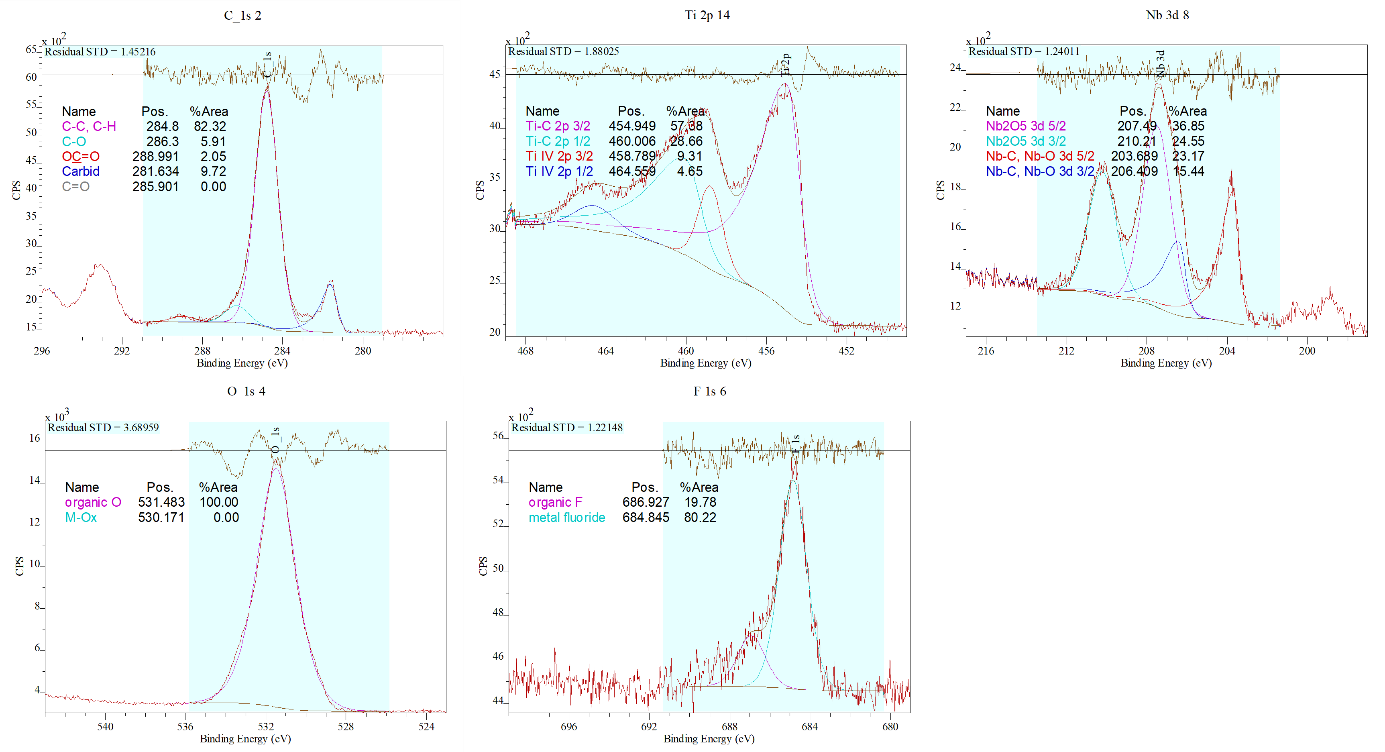


**(a)**


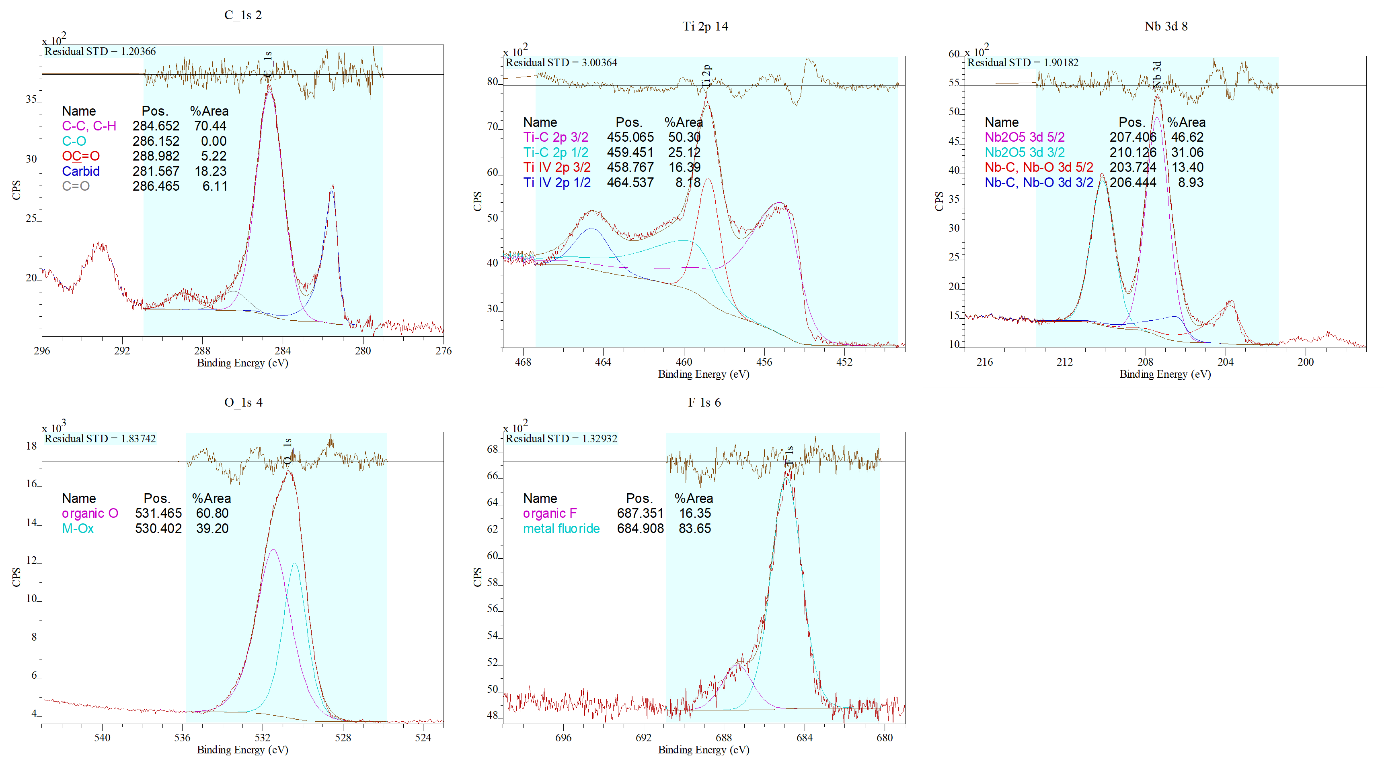


**(b)**

Figure S10: XPS survey of a) the reference (no tribological test) hybrid coating, and b) the wear track.

Table S3: Quantification of XPS survey over two scanned areas and oxide/carbide ratio.

[1] W. Lv, J. Zhu, F. Wang, und Y. Fang, „Facile synthesis and electrochemical performance of TiO2 nanowires/Ti3C2 composite“, *J. Mater. Sci. Mater. Electron.*, Bd. 29, Nr. 6, S. 4881–4887, März 2018, doi: 10.1007/s10854-017-8446-5.

[2] K. Allen-Perry *u. a.*, „Tuning the Magnetic Properties of Two-Dimensional MXenes by Chemical Etching“, *Materials*, Bd. 14, Nr. 3, S. 694, Feb. 2021, doi: 10.3390/ma14030694.

[3] H. Ashraf *u. a.*, „A CoFe2O4/Nb2C-MXene-Modified Anode Improved the Performance Characteristics of a Microbial Fuel Cell in Terms of Bioelectricity Generation and Water Treatment“, *Catalysts*, Bd. 14, Nr. 12, S. 862, Nov. 2024, doi: 10.3390/catal14120862.

[4] L. Du, H. Duan, Q. Xia, C. Jiang, Y. Yan, und S. Wu, „Hybrid Charge‐Storage Route to Nb_2_ CT_x_ MXene as Anode for Sodium‐Ion Batteries“, *ChemistrySelect*, Bd. 5, Nr. 3, S. 1186–1192, Jan. 2020, doi: 10.1002/slct.201903888.

[5] A. Rosenkranz *u. a.*, „Multi-layer Ti3C2Tx-nanoparticles (MXenes) as solid lubricants – Role of surface terminations and intercalated water“, *Appl. Surf. Sci.*, Bd. 494, S. 13–21, Nov. 2019, doi: 10.1016/j.apsusc.2019.07.171.

[6] M. Chhattal *u. a.*, „Solid lubrication performance of Ti2CTx coatings with reduced friction and extended durability“, *Tribol. Int.*, Bd. 194, S. 109535, Juni 2024, doi: 10.1016/j.triboint.2024.109535.

[7] A. Rosenkranz *u. a.*, „Solid-lubrication performance of Ti3C2Tx - Effect of tribo-chemistry and exfoliation“, *Mater. Today Nano*, Bd. 25, S. 100464, März 2024, doi: 10.1016/j.mtnano.2024.100464.

[8] P. G. Grützmacher *u. a.*, „Superior Wear-Resistance of Ti3C2Tx Multilayer Coatings“, *ACS Nano*, Bd. 15, Nr. 5, S. 8216–8224, Mai 2021, doi: 10.1021/acsnano.1c01555.

[9] D. F. Zambrano-Mera *u. a.*, „Solid lubrication performance of sandwich Ti3C2Tx-MoS2 composite coatings“, *Appl. Surf. Sci.*, Bd. 640, S. 158295, Dez. 2023, doi: 10.1016/j.apsusc.2023.158295.

[10] G. Boidi *u. a.*, „Solid lubrication performance of hybrid Ti3C2Tx/MoS2 coatings“, *Carbon*, Bd. 225, S. 119067, Mai 2024, doi: 10.1016/j.carbon.2024.119067.

[11] S. Huang, K. C. Mutyala, A. V. Sumant, und V. N. Mochalin, „Achieving superlubricity with 2D transition metal carbides (MXenes) and MXene/graphene coatings“, *Mater. Today Adv.*, Bd. 9, S. 100133, März 2021, doi: 10.1016/j.mtadv.2021.100133.

[12] J. F. Curry *u. a.*, „Highly Oriented MoS2 Coatings: Tribology and Environmental Stability“, *Tribol. Lett.*, Bd. 64, Nr. 1, S. 11, Sep. 2016, doi: 10.1007/s11249-016-0745-0.
